# Early *Leishmania* infectivity depends on miR-372/373/520d family-mediated reprogramming of polyamines metabolism in THP-1-derived macrophages

**DOI:** 10.1038/s41598-024-51511-y

**Published:** 2024-01-10

**Authors:** J. C. R. Fernandes, S. M. Muxel, M. A. López-Gonzálvez, C. Barbas, L. M. Floeter-Winter

**Affiliations:** 1 Instituto de Medicina Tropical da Faculdade de Medicina, Universidade de São Paulo (IMT-FMUSP), São Paulo, Brazil; 2 Instituto de Biociências, Universidade de São Paulo (IB-USP), São Paulo, Brazil; 3 Instituto de Ciências Biomédicas, Universidade de São Paulo (ICB-USP), São Paulo, Brazil; 4 Centre for Metabolomics and Bioanalysis (CEMBIO), Department of Chemistry and Biochemistry, Facultad de Farmacia, Universidad San Pablo-CEU, CEU Universities, Urbanización Montepríncipe, 28660 Boadilla del Monte, Madrid Spain

**Keywords:** Innate immune cells, Metabolomics, miRNAs, Parasitology

## Abstract

*Leishmania amazonensis* is a protozoan that primarily causes cutaneous leishmaniasis in humans. The parasite relies on the amino acid arginine to survive within macrophages and establish infection, since it is a precursor for producing polyamines. On the other hand, arginine can be metabolized via nitric oxide synthase 2 (NOS2) to produce the microbicidal molecule nitric oxide (NO), although this mechanism does not apply to human macrophages since they lack NOS2 activity. MicroRNAs (miRNAs) are small noncoding RNAs that regulate gene expression at posttranscriptional levels. Our previous work showed that mmu-miR-294 targets *Nos2* favoring *Leishmania* survival in murine macrophages. Here, we demonstrate that human macrophages upregulate the hsa-miR-372, hsa-miR-373, and hsa-miR-520d, which present the same seed sequence as the murine mmu-miR-294. Inhibition of the miR-372 impaired *Leishmania* survival in THP-1 macrophages and the effect was further enhanced with combinatorial inhibition of the miR-372/373/520d family, pointing to a cooperative mechanism. However, this reduction in survival is not caused by miRNA-targeting of NOS2, since the seed-binding motif found in mice is not conserved in the human 3′UTR. Instead, we showed the miR-372/373/520d family targeting the macrophage’s main arginine transporter SLC7A2/CAT2 during infection. Arginine-related metabolism was markedly altered in response to infection and miRNA inhibition, as measured by Mass Spectrometry-based metabolomics. We found that *Leishmania* infection upregulates polyamines production in macrophages, as opposed to simultaneous inhibition of miR-372/373/520d, which decreased putrescine and spermine levels compared to the negative control. Overall, our study demonstrates miRNA-dependent modulation of polyamines production, establishing permissive conditions for intracellular parasite survival. Although the effector mechanisms causing host cell immunometabolic adaptations involve various parasite and host-derived signals, our findings suggest that the miR-372/373/520d family may represent a potential target for the development of new therapeutic strategies against cutaneous leishmaniasis.

## Introduction

Leishmaniasis is a widespread disease affecting an estimated annual count of 0.7 to 1 million individuals. The disease manifests in various clinical forms, such as cutaneous, mucocutaneous, and visceral caused by approximately 20 distinct *Leishmania* species^1^. *Leishmania amazonensis* causes cutaneous leishmaniasis^2^.

Following the transmission of *Leishmania* promastigotes by infected sandflies, the parasite thrives within macrophages by exploiting the host cell environment, which undergoes extensive metabolic shift essential for infection establishment and evasion of immune responses^3–6^.

l-arginine metabolism was extensively studied in both macrophage and *Leishmania*^6^*.* The parasite relies on amino acid permease 3 (*aap3*) for arginine uptake, with arginase activity being essential for the synthesis of ornithine and polyamines in promastigotes^7–11^, while intracellular amastigotes exploit host-derived polyamines^12^. In parallel, macrophage arginine uptake occurs via cationic amino acid (CAT) transporters, specifically CAT1 (SLC7A1) and CAT2 (SLC7A2), which are essential for substrate supply in immune response mounting^13,14^.

*Leishmania* infection can switch macrophage’s l-arginine metabolism to produce polyamines, favoring parasite replication, as demonstrated in vitro and in vivo in the murine model^6^. Since in human monocytes and macrophages polarizing stimuli fail to induce significant nitric oxide synthase 2 (NOS2) or arginase 1 (ARG1) activity^15,16^, the mechanisms by which *Leishmania* controls human macrophage metabolism remain elusive.

MicroRNAs (miRNAs) are small post-transcriptional regulatory non-coding RNAs. The decay of target mRNAs is mediated by miRNA binding to complementary sequences in the 3′UTR region, particularly by the seed region, which is conserved within miRNA family members^17^. These miRNAs also contribute to the metabolic shift observed in human monocytes and macrophages upon exposure to inflammatory stimuli^18,19^.

Several miRNAs have been described as important regulators of the immune response^20^, and *Leishmania* infection can alter the expression profile of these molecules in the host macrophage in murine models and human cells, as demonstrated in *L. amazonensis*^21–23^, *L. braziliensis*^24^, *L. major*^25–27^, *L. donovani*^25,28–31^, and *L. infantum chagasi*^32,33^.

Our study aimed to explore the impact of *L. amazonensis* infection on human miRNAs in THP-1 macrophages and unravel their involvement in orchestrating metabolic rewiring. To achieve this, we examined the effects of altered miRNA expression using metabolomics. Our findings demonstrate the crucial role of hsa-miR-372/373/520 miRNAs in facilitating macrophage metabolic adaptation.

## Materials and methods

### THP-1 culture and differentiation

The THP-1 human monocytic leukemia cell line was purchased from ATCC (American Type Culture Collection, VA, USA, TIB-202™). Cells are non-adherent and were maintained in filtered T75 culture flasks in Roswell Park Memorial Institute (RPMI) 1640 medium (Gibco BRL Products, Grand Island, NY, USA) supplemented with 2 mM glutamine, penicillin (20 U/mL), streptomycin (20 μg/mL), sodium bicarbonate (2.3 g/L) and 10% fetal bovine serum (FBS, Gibco) at 34 ºC and 5% CO_2._ Cells were cultivated at a density of approximately 3–8 × 10^5^ cells/mL to maintain exponential growth up to passage 25 (~ 3 months) and were tested and verified as mycoplasma-free^34^.

Cell number was calculated using Trypan blue exclusion (1:1 cells: Trypan blue, Sigma) to ensure that > 95% were viable. After plating cells in 24-wells (1 × 10^6^) or 6-wells (4 × 10^6^) culture dishes, THP-1 monocytes were treated with 30 ng/mL (48 nM) phorbol 12-myristate 13-acetate (PMA) for 72 h followed by a 72 h incubation with fresh RPMI 1640 10% FBS. After differentiation, cells became adherent and expressed CD11b^+^ (Invitrogen, PE-Cyanine5 M1/70).

### Parasite culture

*Leishmania amazonensis* (MHOM/BR/1973/M2269) promastigotes were maintained at 25 ºC in M199 medium (Invitrogen, Grand Island, NY, USA) supplemented with 10% heat-inactivated fetal bovine serum (FBS, Invitrogen), 5 ppm hemin, 100 μM adenine, 50 U penicillin (Invitrogen), 50 μg/mL streptomycin (Life Technologies, Carlsbad, CA, USA) and 40 mM Hepes–NaOH/12 mM NaHCO_3_ buffer (pH 6.85). Arginase knockout *L. amazonensis* (*La-arg*^*−*^) was cultured in M199 (Invitrogen) supplemented with 100 nM putrescine, 50 mg/mL hygromycin, and 10 mg/mL puromycin as described in^10^. Parasites were maintained at maximum at the 4th passage with an initial parasite load of 5 × 10^5^ parasites, which achieves the stationary growth phase after 7 days.

### In vitro macrophage infection

After differentiation, macrophages were exposed to *L. amazonensis* wildtype (*La-*WT) or arginase knockout (*La-arg*^*−*^) promastigotes at the stationary growth phase (7th day of culture) at 5:1 MOI (multiplicity of infection) and incubated for 4 h at 34 ºC and 5% CO_2_. Non-phagocytized parasites were removed with double washing with 1X PBS, and cells were collected for early infection analysis (4 h) or received fresh RPMI 1640 + 10% FBS medium for further collection (24 h).

For infectivity assays, parasites were labeled before infection using 5 μM CFDA SE Vybrant CFDA SE Cell Tracer (CFSE, Invitrogen) diluted in 1 mL PBS for 1 × 10^8^ parasites for 20 min at 25 ºC, followed by washing with RPMI 10% SFB. To assess infectivity using flow cytometry, cells were detached using PBS/EDTA 1 mM and fixed using PBS/paraformaldehyde (PFA) 1%. The content was then centrifuged and resuspended in 25 µL de PBS 1X. The data were acquired in an imaging flow cytometer FlowSight^®^. Analysis was performed in IDEAS software using the *Wizard Spot Count* tool to determine the number of internalized parasites per macrophage by merging macrophage images in the light channel and CFSE-labeled parasites (Suppl. Fig. 1).

### Total RNA extraction

Macrophages were lysed directly in 6-well plates using 750 µL of QIAzol lysis reagent (Qiagen) for 10 min, followed by addition of 250 µL of PBS and immediately stored in − 80 °C after collection. The RNA extraction was performed using the miRNeasy Mini Kit (Qiagen). Quantitative and qualitative analysis was performed using the NanoDrop spectrophotometer. The extracted RNA purity was assessed by the absorbance ratio A260/A280, which must be around 2. The samples were diluted to 1 µg/10 µL to use in the reverse transcription reactions.

### Reverse transcription and RT-qPCR for miRNAs

The reverse transcription of mature miRNAs was performed using the “miScript II RT Kit” (Qiagen) from 2 µg of total RNA following the manufacturer’s instructions. The miRNA-cDNA was diluted in 150 µL of RNase-free water and stored at 20 °C. We quantified 84 miRNAs using the “Human Inflammatory Response & Autoimmunity miRNA PCR Array: MIHS-105Z” and the “miScript SYBR PCR Kit” (Qiagen). Reactions were performed in a QuantStudio 3 (Applied Biosystems) thermocycler for 95 °C for 15 s followed by 40 cycles of 94 °C for 15 s, 55 °C for 30 s, and 70 °C for 30 s. Each miRNA had its C_t_ expression values subtracted from the medium C_t_ of endogenous control genes (SNORD61, SNORD68, SNORD72, SNORD95, SNORD96A, and RNU6B) to obtain ΔC_t_ values. The ΔΔC_t_ was calculated by subtracting the ΔC_t_ from infected macrophages from the ΔC_t_ of non-infected macrophages. The log2FC (log2 fold change) was calculated as the log_2_(2^−ΔΔCt^), and differentially expressed miRNAs were considered when Log2FC > 1 or < − 1. miRNA expression levels were further validated using miScript Primer Assay (Qiagen) normalized with SNORD95 expression.

Primers for the miR-372 and miR-373 precursors, both pri-miR and pre-miR, were designed based on miRNA structure^35^ (Table 1).

**Table 1:** Primers for miR-372/373 biogenic intermediates.

Target	Fw	Rev
Pre-miR-372/272	GATTGGGTGAGGGGGCG	GACGCTCAAATGTCGCAGC
Pre-miR-372	GGCCTCAAATGTGGAGCACTA	GACGCTCAAATGTCGCAGC
Pre-miR-373	TACTCAAAATGGGGGCGCTT	ACACCCCAAAATCGAAGCACT

Pre: precursor, Pri: primary, Fw: forward primer, Rev, reverse primer.

### Reverse transcription and RT-qPCR for mRNAs

Reverse transcription for cDNA-mRNA synthesis was performed from 2 µg of total RNA, 3 µg of Random Hexam Primer, and 2 μL dNTPs (10 μM) with RNase-free water q.s.p 24 μL, incubated for 5 min at 70 °C at the thermocycler (Mastercycler Gradient, Eppendorf). While the tubes were in the thermocycler at 8 °C, it was added 8 μL of the 5× reaction buffer (250 mM TrisHCl (pH 8.3); 250 mM KCl; 20 mM MgCl_2_ e 50 mM DTT), 2 μL of the RevertAid Reverse Transcriptase (Thermo Scientific, Finland), or RNase-free water in the control reaction, and 2 μL of RiboLock RNase Inhibitor (Thermo Scientific). The program continued in the subsequent incubations: 37 °C–5 min, 25 °C–10 min, 42 °C–60 min, and 70 °C–10 min.

The primers were designed using the Primer-BLAST tool from the *National Center for Biotechnology Information* (NCBI) from reference sequences (RefSeq) of the genes of interest, by flanking an intronic sequence to guarantee that genomic DNA will not be amplified (Table 2).

**Table 2:** Oligonucleotides used for quantification of genes of interest.

Target	Fw	Rev
NOS2	AAGCCTACCCCTCCAGATGA	CTTTGTTACCGCTTCCACCC
ARG1	AAGGATTATGGGGACCTGCC	CGCTTGCTTTTCCCACAGAC
CAT1	CTCATTTAAGGTTCCCTTCCTGC	CAGCATCCACACAGCAAACC
CAT2	CCTTATGGCTTTACGGGAACG	TTCTGGGGATTCCGAACTTCTT
ODC1	GACCACGCACATGTAAAGCC	CAATCCGATCGAGGCCATCA
AMD1	GAGTGAGCTTGACCCAGCAG	TCACGAATTCCACTCTCACGA
SRM	ACAGCCCTCAAGGAAGATGGT	GGAACAGGGACTGGCAGAACT
SMS	CGAAAAACGTGTGGCGATGT	TCCCTTCTTTGGCGTACCTC
GAPDH	GGCAAATTCAACGGCACACT	CCTTTTGGCTCCACCCTTCA

For the RT-qPCR, the reaction was prepared using 5 μL of 10× diluted cDNA/mRNA, 2× Maxima SYBR Green qPCR Master Mix (Thermo Scientific), 0.2 μM from each primer, and RNase-free water for a final volume of 20 μL.

The reaction was performed in a PikoReal 96 RealTime PCR System (Thermo Scientific) thermocycler, by setting 50 °C–2 min, 94 °C–10 min, and 40 cycles of 94 °C–30 s, and 61 °C–30 s. After threshold adjustments, the C_t_ values were normalized using the endogenous control GAPDH. The ΔΔC_t_ was obtained by the difference from the ΔC_t_ of the infected macrophage and the ΔC_t_ of non-infected macrophages or, in the miRNA inhibition assays, the difference of ΔC_t_ from non-treated infected macrophage and the ΔC_t_ from infected macrophages treated with negative control or miRNA inhibitors. The LOG2FC (log2 *Fold change*) values were obtained by calculating the log_2_(2^−ΔΔCt^).

### Transfection of miRNA inhibitors and mimics

After THP-1 macrophage differentiation, we transfected 50 nM of hsa-miR-372-3p, hsa-miR-373-3p, and/or hsa-miR-520d-3p mimics or inhibitors or the positive and negative controls (miRVana, Thermo Scientific), previously incubated with the HiPerfect Transfection Reagent (Qiagen). Infection was performed 24 h after oligonucleotide transfection.

### Sample preparation for metabolomics and metabolite extraction

We prepared 4 groups for metabolomics analysis: NI: non-infected macrophages, *La*: THP-1 macrophages infected with *L. amazonensis*, miR: *La*-infected THP-1 transfected with miR-372/373/520d inhibitors, and NC: *La*-infected THP-1 transfected with scrambled oligonucleotide (negative control). After 4 h of infection, non-phagocyted promastigotes were washed with PBS and the pellets were stored at -80 ºC. Sample preparation and collection were performed in the Laboratory of Physiology of Trypanosomatids (IB-USP) and were shipped to Universidad CEU San Pablo in dry ice through the World Courier logistics company to follow metabolomics analysis procedures. The temperature was stable during transportation.

At Universidad CEU San Pablo, the frozen dried pellets from 4 × 10^6^ macrophages were first vortexed with 350 µL cold methanol/water (4:1) following 4 cycles of freeze/unfreeze in Liquid N_2_/ice, 1 min each, and processing in the TissueLyzer LT (Qiagen, Germany) with 25 mg of glass beads (710–1180 µM, G1152, Sigma Aldrich, Germany) for 10 min under 50 Hz with a 1-min interval with sample on ice. The samples were centrifuged (10 min, 15,700×*g*, 4 °C) and 300 µL of the supernatant was transferred to a new Eppendorf tube. Out of this volume, an 80 µL aliquot was reserved in chromacol vials for analysis at the HILIC-LC–MS instrument. The remaining supernatant was dried in SpeedVac SPD121P (Thermo Fisher Scientific, Waltham, MA.) at 35 °C for 2.5 h. The solid residue was resuspended in 110 µL of a solution containing 0.025 M formic acid containing the internal standards (IS) (0.2 mM of methionine sulfone, 0.2 mM 2-morpholinoethanesulfonic acid (MES), and 1 mM paracetamol) and mixed for 15 min in a vortex. After centrifugation (15,700×*g* for 10 min at 4 °C), the quality control (QC) samples were prepared by pooling equal volumes (5 µL) of each sample. Finally, the QC mixture and 90 µL of each sample’s supernatant were transferred into polypropylene vials (Agilent Technology, Waldbronn, Germany). Samples were stored at 4 °C before each run, and remaining sample was stored for further validation experiments. The samples were placed in a randomized order in CE and LC autosamplers. We originally had 18 samples for each condition, but some exclusions were made in each run due to sample loss or unsuccessful runs.

### CE-ESI-TOF/MS metabolomics

The CE-ESI(+)-TOF/MS system used comprises a capillary electrophoresis system (7100 Agilent Technologies) coupled to a TOF Mass Spectrometer (6230 Agilent Technologies) equipped with an ESI source (G1607 Agilent Technologies) and an Agilent 1200 Infinity I Isocratic Pump (Agilent Technologies) to supply the sheath liquid. For CE-ESI(−)-TOF/MS, the TOF/MS system was Agilent 6224, and a platinum needle was used in the ESI source to avoid corrosion when reverse CE polarity was applied. The CE-MS systems were controlled via MassHunter Workstation version B.06.01.

For positive mode CE-ESI(+)-TOF/MS the sheath liquid (6 µL·min^−1^) composition was 200 mL of methanol/water (1/1, v/v) containing 50 µL of two standard reference masses: 0.25 µM purine (m/z 121.0509, C_5_H_4_N_4_) and 0.25 µM HP-0921 (m/z 922.0098, C_18_H_18_O_6_N_3_P_3_F_24_) to allow for correction and high mass accuracy in the MS. MS parameters were as follows: fragmentor 125 V, Skimmer 65 V, octopole 750 V, nebulizer pressure 10 psi, drying gas temperature at 200 °C, and flow rate 10 L·min^−1^ and capillary voltage 3500 V. The data were acquired in positive Dual-ESI mode with a full scan from *m/z* 74 to 1000 Da at a rate of 1.02 scan/s. The separation was performed in a fused silica capillary with 100 cm of total length and 50 μm internal diameter (Agilent Technologies). The run was performed in normal polarity with a background electrolyte that was composed of 1.0 mol·L^−1^ of formic acid solution in 10% methanol (v:v). New capillaries were pre-conditioned with a flush (950 mbars) of NaOH 1.0 mol·L^−1^ for 30 min, followed by MilliQ water for 30 min and background electrolyte for 30 min. The samples were hydro-dynamically injected at 50 mbar for 50 s and stacking was carried out, applying background electrolyte at 100 mbar for 10 s. The separation voltage was 30 kV with 25 mbar of internal pressure and the run time was 40 min^36^.

For negative mode CE-ESI(−)-TOF/MS the sheath liquid (10 µL·min^−1^) was 200 mL of methanol/water (1/1, v/v) containing 50 µL of three standard reference masses: 121.0509 purine (C_5_H_4_N_4_), 112.9856 ammonium trifluoroacetate in acetonitrile:water TFANH_4_ (Agilent) and 922.0098 hexakis(1H, 1H, 3H-tetrafluoropropoxy)phosphazine HP-0921 (C_18_H_18_O_6_N_3_P_3_F_24_). We used the MS parameters: fragmentor 125 V, Skimmer 65 V, octopole 750 V, nebulizer pressure 10 psi, drying gas temperature at 275 °C, and flow rate 10 L·min^−1^ and capillary voltage 2000 V. The data was acquired in negative Dual-ESI mode with a full scan from *m/z* 60 to 1000 at a rate of 1.0 scan/s. Separations were performed using polyvinyl alcohol coated capillary (PVA) (Agilent Technologies) (dimensions: 50 μm ID × 100 cm total length) in reversed polarity. The run was performed in 0.1 mol·L^−1^ of formic acid solution. Before each analysis, the capillaries were conditioned with background electrolyte for 5 min. The samples were hydro-dynamically injected at 50 mbar for 35 s and stacking was carried out, applying background electrolyte at 100 mbar for 10 s. The separation voltage was − 30 kV with 50 mbar of internal pressure and the run time was 40 min^37^.

### HILIC-LC-ESI-QTOF/MS metabolomics

Samples were measured using a liquid chromatography (LC) system (1260 Infinity, Agilent Technologies, Santa Clara, CA, USA), coupled to a quadrupole and a TOF mass spectrometers with ESI (LC-ESI-QTOF-MS), 6545 Agilent Technologies.

Metabolite separation was performed by HILIC on an XBridge Ethylene bridged hybrid Amide 2.5 µm, 2.1 mm × 100 mm (Waters, Milford, MA, USA). The flow was constant at 0.250 mL/min and the column was kept at 25 °C and 50 °C in positive and negative mode respectively. For positive mode, the mobile phases consisted of 10 mM ammonium formate, 2.5 mM of Infinitylab deactivator additive (Agilent Technologies), and 0.1% formic acid diluted in water (phase A) and in water and acetonitrile (Fischer Scientific, UK) (H_2_O/ACN, 10:90, v/v) (phase B). For negative mode, the mobile phases consisted of 10 mM ammonium acetate and 2.5 mM of Infinitylab deactivator additive (Agilent Technologies) diluted in water (phase A) and water and acetonitrile (H_2_O/ACN, 15:85, v/v) (phase B). In the negative mode, we used a stacking of 8 μL of ACN in both leading and terminating bands. The gradient consisted of a mix of mobile phases A and B as described in^38^.

The samples maintained at 4 °C and 3 μL of the samples were either directly injected in the mobile phase flow in positive mode or stacked in ACN using sandwiching between two bands of 8 μL in negative mode.

The conditions in the MS were as follows for positive and negative mode: the gas temperature in the ion source was 225 ºC, the flow was 6 and 13 L/min, the pressure in the nebulizer was 40 and 35 psig, the temperature of the sheath gas was 225 and 350 ºC, the flow of the sheath gas was 10 and 12 L/min and the voltage of the capillary was set at 3000 and 3500 V respectively. The nozzle voltage was 0 V, the fragmentor was set at 125 V, the voltage in the skimmer was 65 V and the Octopole was 450. Data were acquired from 70 to 1100 m/z in centroid mode at 3 scans per second. Five iterative MS/MS fragmentations were performed on a QC sample at both positive and negative ionization modes at 20 and 40 V for the metabolite annotation process.

### Metabolomics data processing and statistical analysis

The raw mass chromatogram was aligned using IS migration times as parameters using the Mass Hunter Profinder software 10.0 (Agilent Technologies), followed by peak deconvolution and integration. The final matrix containing the peak area for each feature was processed including missing values using our in-house K-nearest neighbors’ script for MATLAB (MathWorks, R2018b) and the values were normalized to correct intra-batch effects by QC support vector regression correction (QC-SVRC)^39^ to correct instrument variations based on QC sample variability for data of CE-ESI(+)-TOF/MS and HILIC-ESI-QTOF-MS data. The total useful signal (TUS) values were used for the normalization of the CE-ESI(−)-TOF/MS data. After correction, data were filtered by the relative standard deviation (RSD) < 30% in QC samples. Metabolite identification was performed from metabolite exact mass and RMT^37,40^ with the CEU Mass database^41^, using a maximum of 20 ppm error. For the final annotations, we also combined information on ion-source fragmentation patterns for TOF/MS data and MS/MS fragmentation for QTOF/MS, allowing us to achieve the confidence degree (CD) 2, where additional information has been considered (time, adduct formation, in-source fragmentation pattern).

The univariate statistical analysis was performed to compare *La* × NI and miR × NC groups using CEMBIO’s algorithms in MATLAB (Mathworks). Feature normality and homoscedasticity were checked by Kolmogorov and Levene’s tests, respectively, with an alpha of 0.05. Features obeying normality were tested using the t Student’s test with either equal or different variance, while non-normal data were tested using the Mann–Whitney test. Additionally, the FDR-corrected *p*-values (Benjamin-Hochberg) were considered significant when < 0.05.

The multivariate statistical analysis (MVDA) was performed in SIMCA 16.0.1 software (Umetrics, Umea, Sweden) for better data comprehension with dimensionality reduction. Principal component analysis (PCA) setting UV scaling (CE-ESI(+)-TOF/MS and HILIC-ESI-QTOF-MS) or Log transformation with Pareto scaling (CE-ESI(−)-TOF/MS) was used to validate the quality of analytical performance. The partial least-squares-discriminant analysis (PLS-DA) and orthogonal partial least-squares-discriminant analysis (OPLS-DA) were carried out for discriminating the variation between the groups, calculating the model’s quality by explained variance (*R*^2^) and predicted variance (Q^2^). Both variable importance in projection (VIP) and partial correlation coefficient (P (corr)) from S-plot or volcano plot data were obtained from the OPLS-DA model, where |P (corr)|> 0.5 and VIP > 1 indicate the importance of the variable in discriminating between both tested groups.

### Simultaneous metabolite and miRNA target enrichment analysis

Reactome enrichment analysis was performed using the available analysis tools (https://reactome.org/PathwayBrowser/#TOOL=AT). Significantly changed metabolite names were converted to KEGG codes^42,43^ and submitted. The pathways with FDR- corrected p values < 0.05 were considered enriched. A second submission with putative miR targets was performed in the same platform and genes belonging to each enriched pathway are listed in the results.

### Protein levels

Determination of the CAT2 protein level was performed using BD Accuri ™ C6 flow cytometry and FlowJo software. After macrophage differentiation, infection, or miRNA mimics treatment, cells were detached with 100 μL of PBS/EDTA 1 mM and transferred to 96-well plates, centrifuged, and fixed with 1% PFA. Permeabilization was performed using 100 μL of BD Cytofix/Cytoperm for 20 min and washing using the BD Perm/Wash, followed by staining with the primary antibody anti-CAT2 (Santa Cruz CA, USA, sc-87038) in 1:100 concentration at 4 ºC overnight, followed by the secondary antibody Donkey anti-Goat IgG (Invitrogen PA1-29953) in the concentration 1:200 for 2 h at room temperature. After that, the cells were washed using the BD Perm/Wash, resuspended at 100 μL of PBS, and analyzed in the cytometer.

### pmiRGLO validation

The pmirGLO *Dual-Luciferase miRNA Target Expression Vector* (Promega) containing the 3′UTR sequences of the SLC7A2 (CAT2) gene were constructed by cloning the miRNA-biding sequences, complementary to the seed, and its surroundings, either the standard sequences or mutated sequences (Table 3) after the Firefly luciferase gene. RAW 264.7 (TIB-71, ATCC) macrophages (1 × 10^6^) were transfected using 5 μg of the pmiRGLO vector using the Transfection reagent FuGene HD (Promega) and treated with 50 nM of miRNA mimics. After 24 h of transfection, macrophages were detached using PBS 1 mM EDTA, washed with PBS 1X, diluted in RPMI medium, and transferred to 96-well black microplates with transparent bottoms. The luciferase reaction was performed using the Dual-Glo^®^ Luciferase Assay (Promega), following the manufacturer’s instructions, and read in the GloMax^®^ luminometer (Promega).

**Table 3:** Single-stranded DNA sequences for annealing and cloning in the pmiRGLO vector.

3′ UTR	*Seed*	Orientation
Sequence		
CAT2.2	3947–3953	Sense
CTAGCGGCCGCCCCCATAAATTGTGTAGCACTTTT		
CAT2.2	3947–3953	Antisense
CTAGAAAAGTGCTACACAATTTATGGGGGCGGCCGCTAGAGCT		
CAT2.2 mutated	3947–3953*	Sense
CTAGCGGCCGCCCCCATAAATTGTGTAGACTTTT		
CAT2.2 mutated	3947–3953*	Antisense
CTAGAAAAGTCTACACAATTTATGGGGGCGGCCGCTAGAGCT		
CAT2.1	1327–1333	Sense
CTAGCGGCCGCCATGATGACAGAGGGAGCACTTGT		
CAT2.1	1327–1333	Antisense
CTAGACAAGTGCTCCCTCTGTCATCATGGCGGCCGCTAGAGCT		
CAT2.1 mutated	1327–1333*	Sense
CTAGCGGCCGCCATGATGACAGAGGGAGACTTGT		
CAT2.1 mutated	1327–1333*	Antisense
CTAGACAAGTCTCCCTCTGTCATCATGGCGGCCGCTAGAGCT		

* Oligonucleotide mutated.

### Statistical analysis

Statistical analysis of all experiments with exception of metabolomics were performed using the GraphPad Prism 9 software (GraphPad Software, Inc., La Jolla, CA, USA) using either t test for comparison of two groups or ANOVA Dunnet’s posthoc test for multiple comparisons. Data appear as the mean ± standard error of the mean (SEM) of data obtained in at least 2 independent experiments and the p values are displayed in each figure. Significant changes were considered when p < 0.05.

## Results

### *L. amazonensis* infection upregulates the miRNA family miR-372/373/520d in THP-1 macrophages

**Figure 1.**
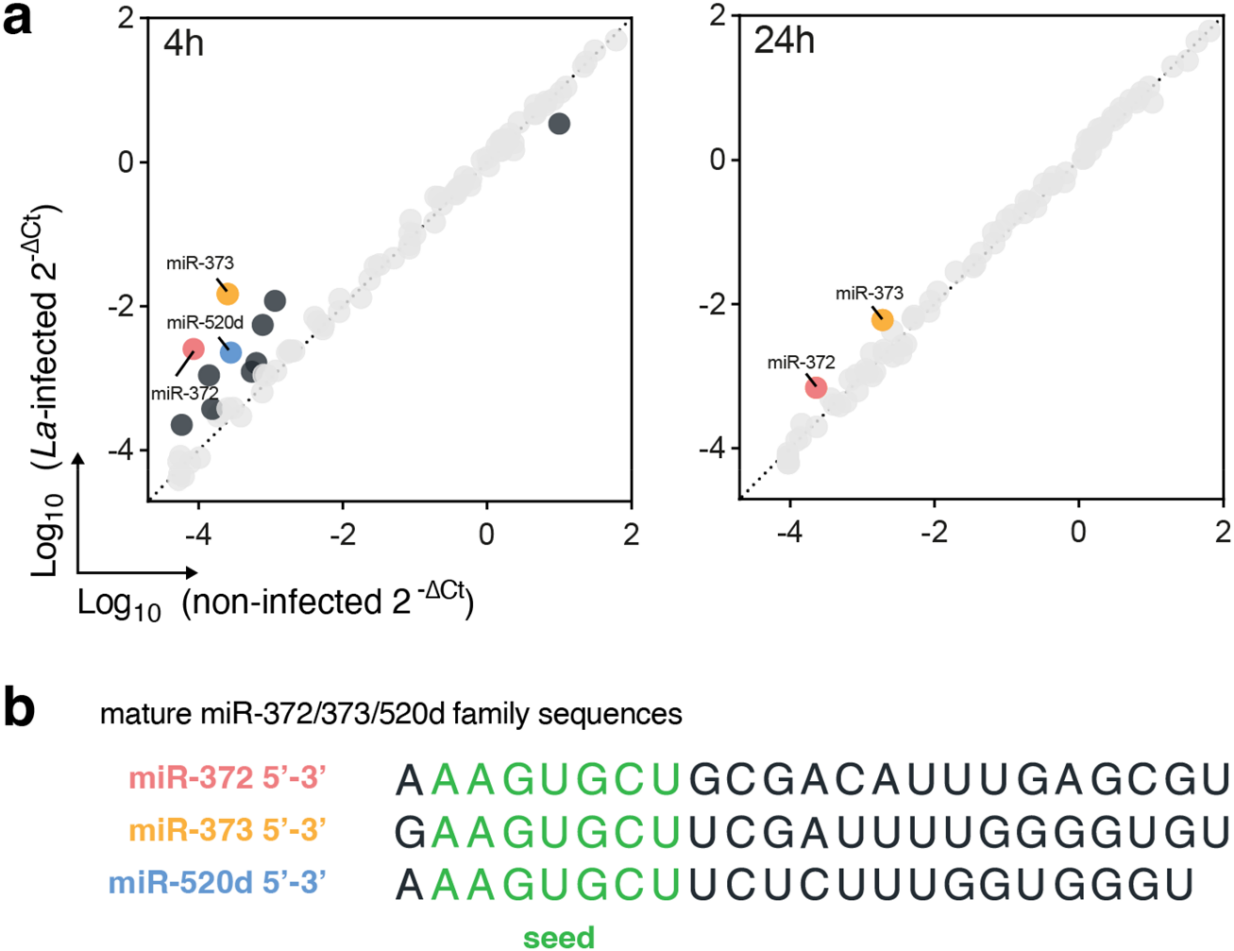
microRNA miR-372/373/520d family is upregulated in THP-1 macrophages infected with *L. amazonensis*. (**a**) Expression of 84 immune response-related miRNAs in THP-1 macrophages after 4 and 24 h of *L. amazonensis (La)* infection assessed by qPCR array highlighting miRNAs with Log2FC > 1 or < − 1 and miRNAs belonging to the same family (N = 4). (**b**) mature miRNA sequences for human miR-372, miR-373 and miR-520d retrieved from miRBase (miRbase.org) highlighting the seed sequences in green.

To investigate if human macrophage microRNAs (miRNAs) are modulated during *L. amazonensis (La)* infection, we used a qPCR array as a starting point, and quantified 84 miRNAs related to immune response in *La*-infected THP-1 macrophages. We identified significant upregulation of miR-202 (log2FC = 3.37, p = 0.0016), miR-300 (log2FC = 1.36, p = 0.0001), miR-372 (log2FC = 4.9, p < 0.0001), miR-373 (log2FC = 5.86, p = 0.0103), miR-381 (log2FC = 2.83, p < 0.0001), miR-520d (log2FC = 3.01, p = 0.0261), miR-543 (log2FC = 2.97, p = 0.0012), and miR-545 (log2FC = 1.95, p = 0.0136) (Suppl. Table 1). We highlighted the miR-372, miR-373, and miR-520d in the scatter plot (Fig. 1a), because these miRNAs contain identical seed sequences (nucleotides 2–8) (Fig. 1b).

We validated the upregulation of miR-372 (4 h: p = 0.0009, 24 h: p = 0.0133), miR-373 (4 h: p < 0.0001, 24 h: p = 0.0005) and miR-520d (4 h: p = 0.0001, 24 h: p = 0.0015) at early time points of infection with *L. amazonensis* by qPCR using specific oligonucleotides (Suppl. Fig. 2a). Although miR-372 and miR-373 primary transcripts are expressed together, we could not see any changes at the pri- and pre-miRNA levels of miR-372/373 (Suppl. Fig. 3).

Also, by using arginase knockout *La (La-arg*^*−*^*)*, we noticed that the upregulation of miR-372/373/520d correlated with infectivity in THP-1 macrophages. In a similar way to wildtype *La-*WT, we found *La-arg*^*−*^ infection can induce miR-372 (p = 0.0004) and miR-373 (p < 0.0001) but failed to induce miR-520d. Infection with *La-arg*^*−*^ also does not sustain miR-372 and miR-373 expression after 24 h of infection. Indeed, *La-arg*^*−*^ reduces miR-373 (p = 0.0007) levels compared to non-infected macrophages at 24 h of infection (Suppl. Fig. 2a). The *La*-WT and *La-arg*^*−*^ models infect similarly at 4 h of infection, but at 24 h *La-arg*^*−*^ cannot grow inside macrophages, while the *La*-WT increases its infectivity, resulting in a lower infection index in the comparison *La-WT* x *La-arg*^*−*^ (p = 0.0005) (Suppl. Fig. 2b). Our data show that absence of parasite’s arginase led to an unstained expression of miR-372/373/520d and reduced infectivity after 24 h of infection, indicating that these miRNAs could contribute to *Leishmania* infectivity.

### Inhibition of miR-372/373/520 family shows cooperative action on early *L. amazonensis* infection

To confirm if the miR-372/373/520 family is critical for *Leishmania* infectivity, we measured *La* infectivity in THP-1 macrophages after transfecting 50 mM of antisense oligonucleotides to inhibit miR-372, miR-373, and miR-520d function and a scramble negative control (NC). As these oligonucleotides disrupt miRNA function without affecting miRNA level (Suppl. Fig. 4b) we confirmed successful inhibition using the same protocol with a commercial positive control (PC) oligonucleotide which de-repressed its known target (Suppl. Fig. 4a).

**Figure 2:**
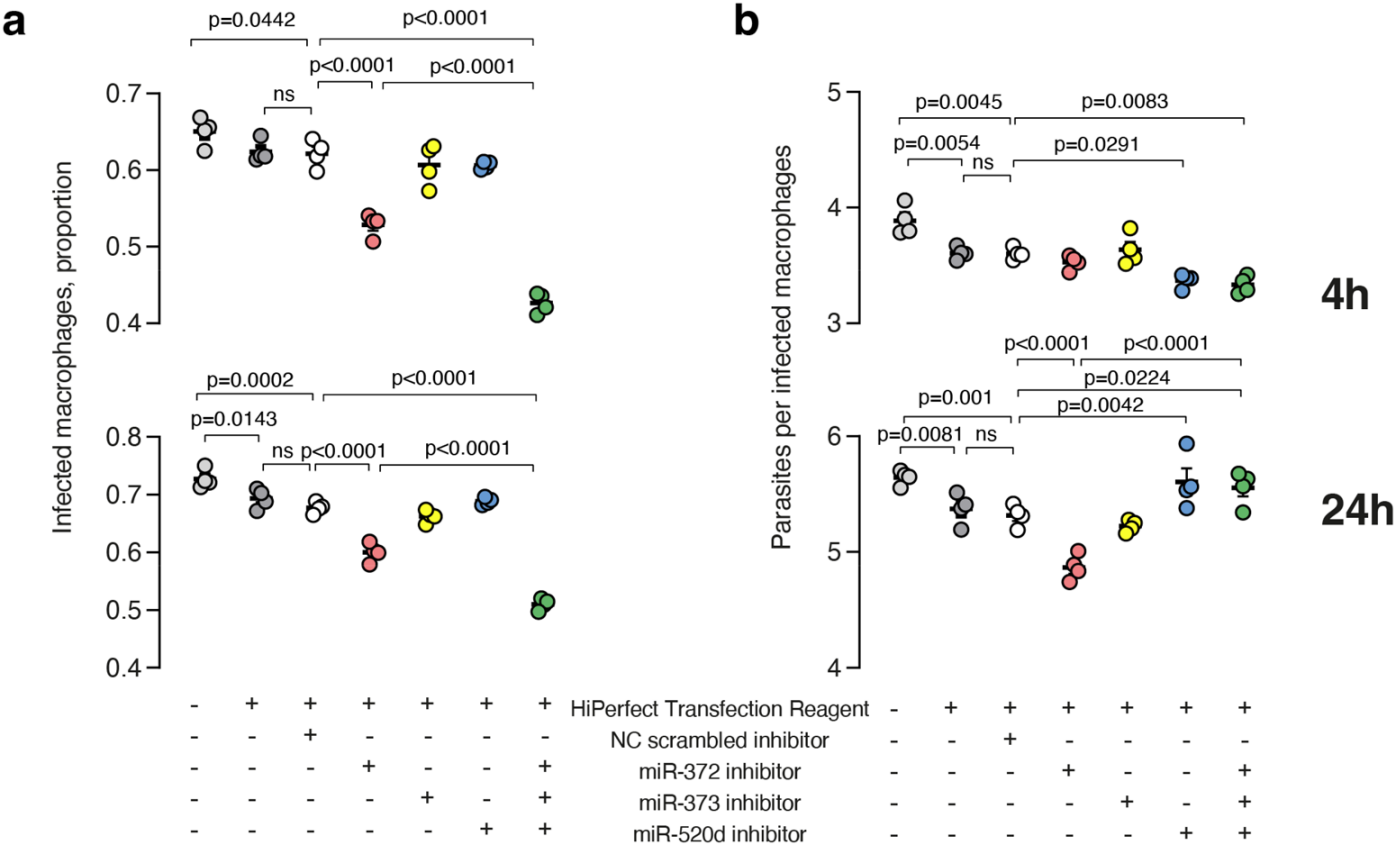
Inhibition of the miR-372/373/520 family impairs *L. amazonensis* infection. (**a**) Proportion of infected macrophages and (**b**) parasites per infected macrophage assessed by imaging flow cytometry of CFSE-labeled parasites. NC: negative control, ns: not significant. Error bars represent means ± sem. P values were assessed by ANOVA Dunnet’s posthoc test and P < 0.05 are depicted in the figure.

Transient inhibition of miR-372, but not miR-373 and miR-520d alone impaired early *Leishmania* infectivity, reducing both the proportion of infected macrophages (4 h: p < 0.0001, 24 h: p < 0.0001) and the number of parasites per infected macrophage (4 h: ns, 24 h: p < 0.0001). Simultaneous depletion of the three miRNAs further reduced the proportion of infected macrophages compared to NC (p < 0.0001) or miR-372 inhibition alone (p < 0.0001). On the other hand, inhibition of miR-372 reduced parasite count per macrophage only after 24 h of infection compared to NC. The antisense oligonucleotide for miR-520d could only reduce the number of parasites per macrophage after 4 h of infection, but, conversely, increased the number of parasites per macrophage after 24 h (p = 0.0291). Also, the transfection of the three inhibitors yielded similar results to the individual miR-520d inhibitor treatment in terms of the analysis of internalized parasites, both at the 4-h and 24-h time points (Fig. 2). It is noteworthy that both treatment with transfection reagent or NC transfection alone significantly reduce infectivity at similar levels (Fig. 2), so all phenotypes were compared with NC instead of non-transfected cells.

The requirement for inhibiting all three miRNAs reveals a coordinating role in maintaining *Leishmania* infectivity, which may reflect compensatory effects by these molecules. However, when only one miRNA was inhibited, we did not observe any upregulation of miRNA expression within the same family (Suppl. Fig. 4b). This finding suggests that the existing levels of mature miRNAs induced during *Leishmania* infection are sufficient to present compensatory functions.

Also, miRNA mimics for the miR-372/373/520 family could increase the percentage of infected macrophages compared to NC at 4 h but did not affect the number of parasites per macrophage (Suppl. Fig. 5).

### *L. amazonensis* infection reprograms arginine metabolism in a miRNA-dependent manner

We used MS-based metabolomics to systematically investigate whether combinatorial miR-372/373/520 miRNA inhibition elicits metabolic changes that could explain reduced infectivity at the phenotype level.

**Figure 3:**
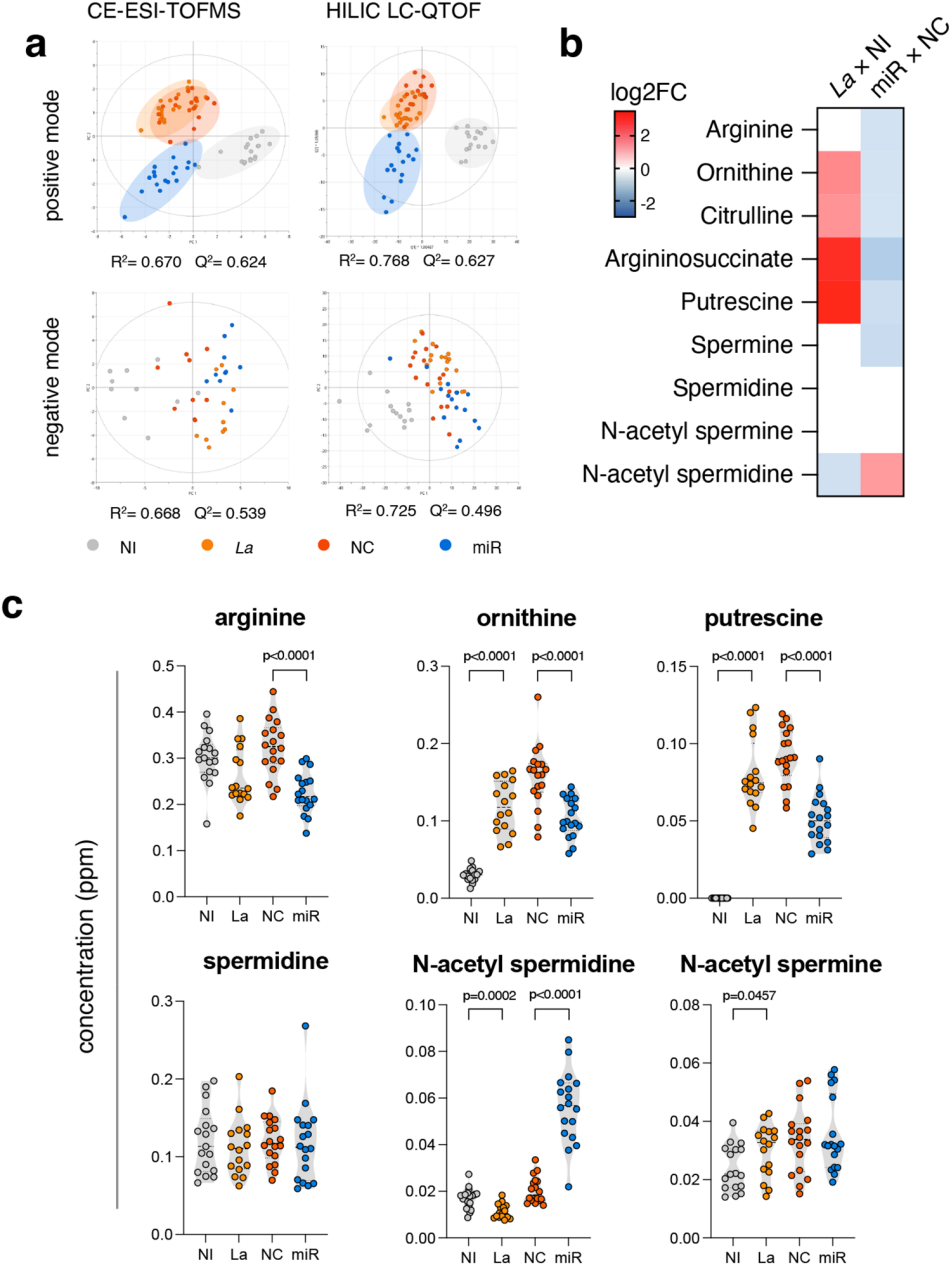
Disruption of miR-372/373/520 action upon *Leishmania* infection significantly alters the metabolome and prevents polyamine production. (**a**) Supervised PLS-DA models for different metabolomics platforms used; (**b**) heatmap of log2FC of significantly changed metabolites from polyamine production pathway; (**c**) arginine metabolism’s metabolites concentration measured with standard curves from analytical standards. NI: non-infected macrophage, *La*: *L. amazonensis*-infected macrophage, NC: *L. amazonensis*-infected macrophage treated with negative control, and miR: *L. amazonensis*-infected macrophage treated with miR-372/373/520d inhibitors. Significant p values (P < 0.05) shown in (**c**) were obtained by two-sample t test.

Unsupervised clustering model PCA-X demonstrated robust reproducibility of QC samples (Suppl. Fig. 6). Under the unsupervised method of analysis, distinct separation was observed between non-infected (NI) samples and the three other groups of samples containing *Leishmania*-infected macrophages (*La*, NC, and miR) (Suppl. Fig. 7a–d). Notably, clear separation and clustering were only observed in the supervised PLS-DA model for CE-ESI(+)-TOF/MS and HILIC-ESI(+)-QTOF-MS data, whereas the data obtained in the negative polarization model did not exhibit such a pattern (Fig. 3a). We further assessed the quality of the data by calculating the model fit with R^2^ values and predictive power with Q^2^ values, indicating good and predictive PLS-DA models. The OPLS-DA (Suppl. Fig. 7e–l) VIP scores were listed in the Suppl. Tables 2 and 3, estimate the importance of each metabolite for group separation.

To elucidate the miRNA targets involved in metabolic adaptations, we searched for metabolites significantly changed in the univariate statistical analysis that were rescued to non-infected macrophage levels upon miRNA inhibition. Among differentially regulated features, we observed that arginine-related metabolism was markedly altered in response to infection and miRNA inhibition. Such a pattern was present for metabolites from the urea cycle (arginine, ornithine, citrulline, and argininosuccinate) and polyamine production (arginine, ornithine, putrescine, and spermine). These metabolites displayed significant upregulation (P < 0.05, FDR-corrected) comparing *La* × NI. In contrast, the miRNA inhibition reduced arginine, ornithine, citrulline, putrescine, and spermine compared to NC levels. In addition, miRNA inhibition resulted in the accumulation of the acetylated form of spermidine (N-Ac SPD) (Fig. 3b,c).

Collectively, this data show reduced polyamine availability upon miRNA inhibition, which could explain reduced infectivity in this condition.

### Putative binding sites for miR-372/373/520 family’s seed are found in the 3′UTR from multiple mRNAs related to arginine metabolism

To gain insights into the potential target genes responsible for the observed metabolic alterations, we retrieved a list of 5206 human genes whose 3′UTR contain miR-372/373/520 family predicted binding sites from the TargetScan Human 7.2 database for in silico analysis (Suppl. Table 4). Our analysis revealed eight significantly enriched pathways from Reactome, which were associated with the metabolism of amino acids and derivatives, translation, tRNA processing, and SLC-mediated transmembrane transport. Upregulation of citrulline, ornithine, argininosuccinic acid, arginine, and adenosine triphosphate led to an enriched urea cycle, while S-adenosylmethionine, N1-acetylspermidine, arginine, ornithine, spermine, and putrescine accounted for production of polyamines.

We found 22 miR-372/373/520 family predicted targets encoding proteins related to both arginine metabolism and urea cycle, respectively, including its main transporters SLC7A1/CAT1 and SLC7A2/CAT2. In the urea cycle, we found the N-acetyl glutamate synthase (NAGS), the aminoacylase (ACY1), the argininosuccinate lyase (ASL), the aspartate-glutamate transporter (SLC25A12), the ornithine translocase (SLC25A15), the mitochondrial arginase 2 (ARG2), the ornithine transcarbamylase (OTC), and synthesis of polyamines—S-adenosylmethionine decarboxylase (AMD1), the peroxisomal N-acetyl-spermine/spermidine oxidase (PAOX), the agmatinase (AGMAT), the antizyme inhibitor 1 (AZIN1) (Suppl. Fig. 8, Table 4 and Suppl. Table 4).

**Table 4:** Significantly enriched pathways from Reactome related to the metabolism of amino acids and derivatives, translation, tRNA processing, and SLC-mediated transmembrane transport. FDR: false discovery rate.

Enriched pathway	FDR	Metabolites (number of molecules;Total molecules)	Predicted miR targets (number of genes; total genes)
Metabolism of amino acids and derivatives			
Glutamate and glutamine metabolism (R-HSA-8964539)	4.75 × 10^–3^	l-Alanine, pyruvic acid, ornithine, l-glutamine, l-glutamate, l-proline, adenosine triphosphate (7;27)	RIMKLA, PYCRL, GLUL (3;42)
Tryptophan catabolism (R-HSA-71240)	1.46 × 10^–6^	l-Kynurenine, l-valine, l-alanine, l-tyrosine, l-isoleucine, pyruvic acid, l-tryptophan, l-phenylalanine, l-leucine, l-histidine (10;34)	AADAT, HAAO, SLC36A4, IDO1 (4;48)
Urea cycle (R-HSA-70635)	1.4 × 10^–2^	Citrulline, ornithine, argininosuccinic acid, l-arginine, adenosine triphosphate (5;22)	NAGS, SLC25A15, ASL, ARG2, ORC1, OTC (6;32)
Metabolism of polyamines (R-HSA-351202)	1.37 × 10^–3^	S-Adenosylmethionine, N1-acetylspermidine, l-arginine, ornithine, spermine, putrescine (6;21)	RPN2, AMD1, PAOX, PSMD1, PSMB2, PSMD5, PSMB1, PSMD12, PSMA4, PSMB5, AGMAT, AZIN1, PSMB9 (13;80)
Translation			
tRNA aminoacylation (R-HAS-379724)	1.8 × 10^–12^	l-Valine, l-arginine, l-alanine, l-tyrosine, l-isoleucine, l-tryptophan, l-asparagine, l-glutamine, l-proline, adenosine triphosphate, l-phenylalanine, l-Leucine, l-lysine, l-histidine (14;26)	SARS, DARS2, KARS, SAR1A, PPA2, RARS2, LARS, AARS2, YARS2, FARSB (10;68)
tRNA processing			
tRNA modification in the nucleus and cytosol (R-HSA-6782315)	4.23 × 10^–2^	S-Adenosylmethionine, pyruvic acid, succinic acid, adenosine triphosphate (4;29)	ADAT2, TYW3, TP53RK, TYW5, KIAA1456, THG1L, ADAT1, TRDMT1 (8;72)
SLC-mediated transmembrane transport			
Transport of inorganic cations/anions and amino acids/oligopeptides (R-HSA-425393)	2.46 × 10^–11^	l-valine, l-arginine, l-alanine, l-tyrosine, pyruvic acid, S-adenosylmethionine, l-isoleucine, ornithine, l-tryptophan, l-asparagine, l-glutamine, l-proline, l-phenylalanine, l-leucine, l-lysine, l-histidine (17;60)	SLC9A7, SLC6A15, CTNS, SLC38A1, SLC15A2, SLC12A6, SLC36A4, SLC17A6, SLC17A5, SLC25A10, SNX12, SLC1A5, LAT2, SLC4A8, SLC9A2 (31;167), SLC7A1^a^, SLC7A2^a^, SLC25A12^a^
Transport of bile salts and organic acids, metal ions and amine compounds (R-HSA-425366)	1,26 × 10^–08^	l-Valine, l-arginine, l-alanine, l-tyrosine, pyruvic acid, l-isoleucine, l-tryptophan, succinic acid, l-asparagine, l-glutamine, l-proline, spermine, l-phenylalanine, l-leucine, l-lysine, l-histidine (16;79)	RUNX1, EMB, SLC30A5, SLC6A9, SLC31A1, SLC6A15, SLC11A1, SLC14A1, SLC16A1, SLC30A6, SLC22A15, SLC14A2, SLC10A6, SLC30A7, SLC16A7, DDI2, SLC39A6, SLC5A7, SLC6A5, SLC5A3, SLC30A10, SLC40A1, SLC44A1, SLC22A1, SLC22A3, SLC47A1, SLC22A5, RHCG, SIT1, (29;165)

^a^Manually curated putative target genes related to the metabolic pathway listed in Target Scan but not listed in Reactome.

**Figure 4:**
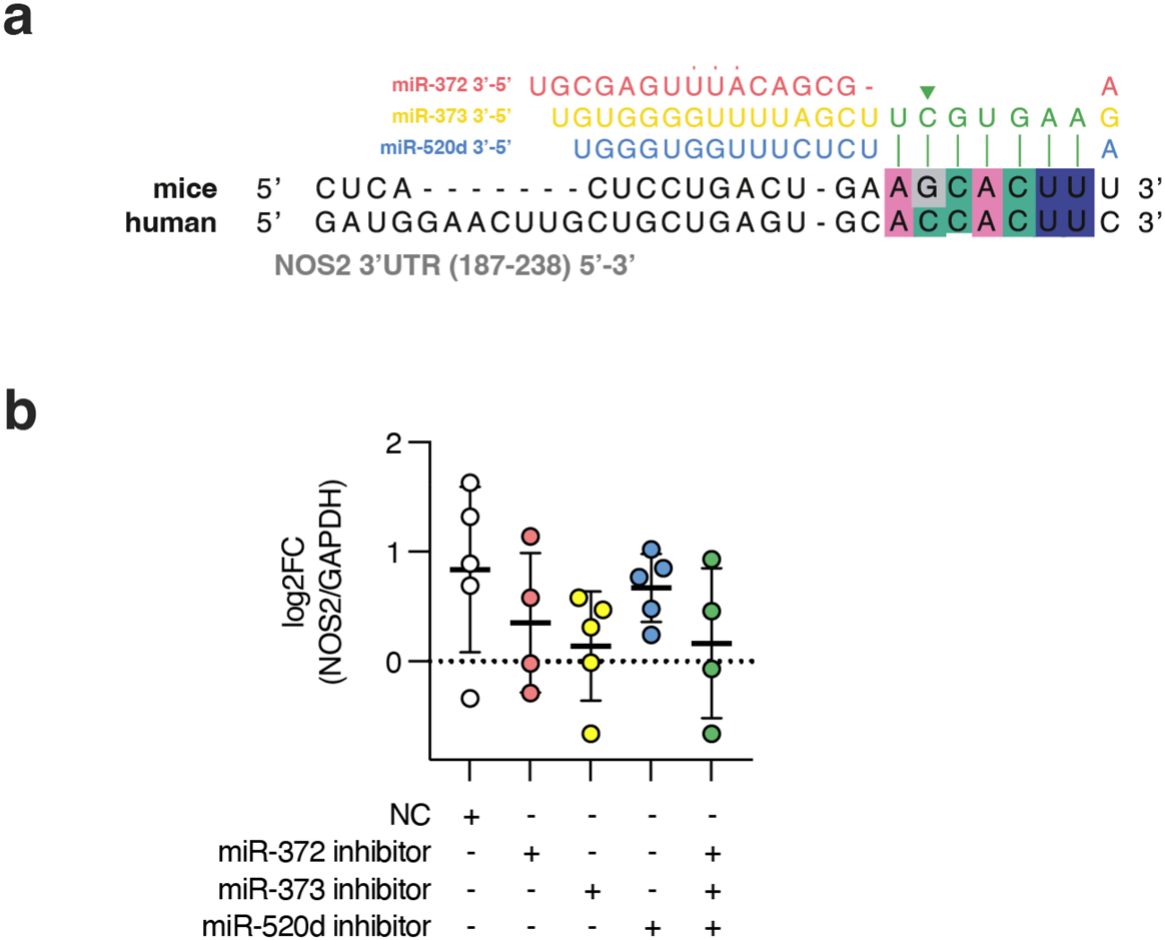
Human NOS2 is not a miR-372/373/520d conserved target. (**a**) sequences of NOS2 3′UTR from human and mice highlighting miR-372/373/520d seed pairing and mutation of the human NOS2 (green arrow head) and (**b**) human NOS2 mRNA quantification upon miRNA inhibition in THP-1 macrophages. NC: negative control. Error bars represent log2FC values ± sem. P values were assessed by one sample t test.

Intriguingly, the human NOS2 3′ UTR is not a predicted target for the miR-372/373/520 family. This observation contrasts with our previous findings in mice, where the homologous murine miRNA miR-294 was shown to target *Nos2* and promote infection. To investigate this discrepancy, we assessed the sequence homology using the TargetScan database and identified a mutation seed binding motif of human NOS2 (Fig. 4a). Subsequently, we performed miRNA inhibition assays, confirming that the miR-372/373/520 family does not target human NOS2 (Fig. 4b).

### Validation of the main arginine transporter CAT2 as a miR-372/373/520 target

To obtain a broader view of arginine metabolism at the mRNA level during *Leishmania* infection, we expanded our analysis to include enzymes that were not predicted as targets of the miR-372/373/520 family. Surprisingly, despite major changes found in metabolomics results, we found no significant alterations in the mRNA levels of arginine metabolism-related genes SLC7A1/CAT1, SLC7A2/CAT2, NOS2, ARG1, ODC, SMS, SRM, and ADOMET after *L. amazonensis* infection (Suppl. Fig. 9).

**Figure 5:**
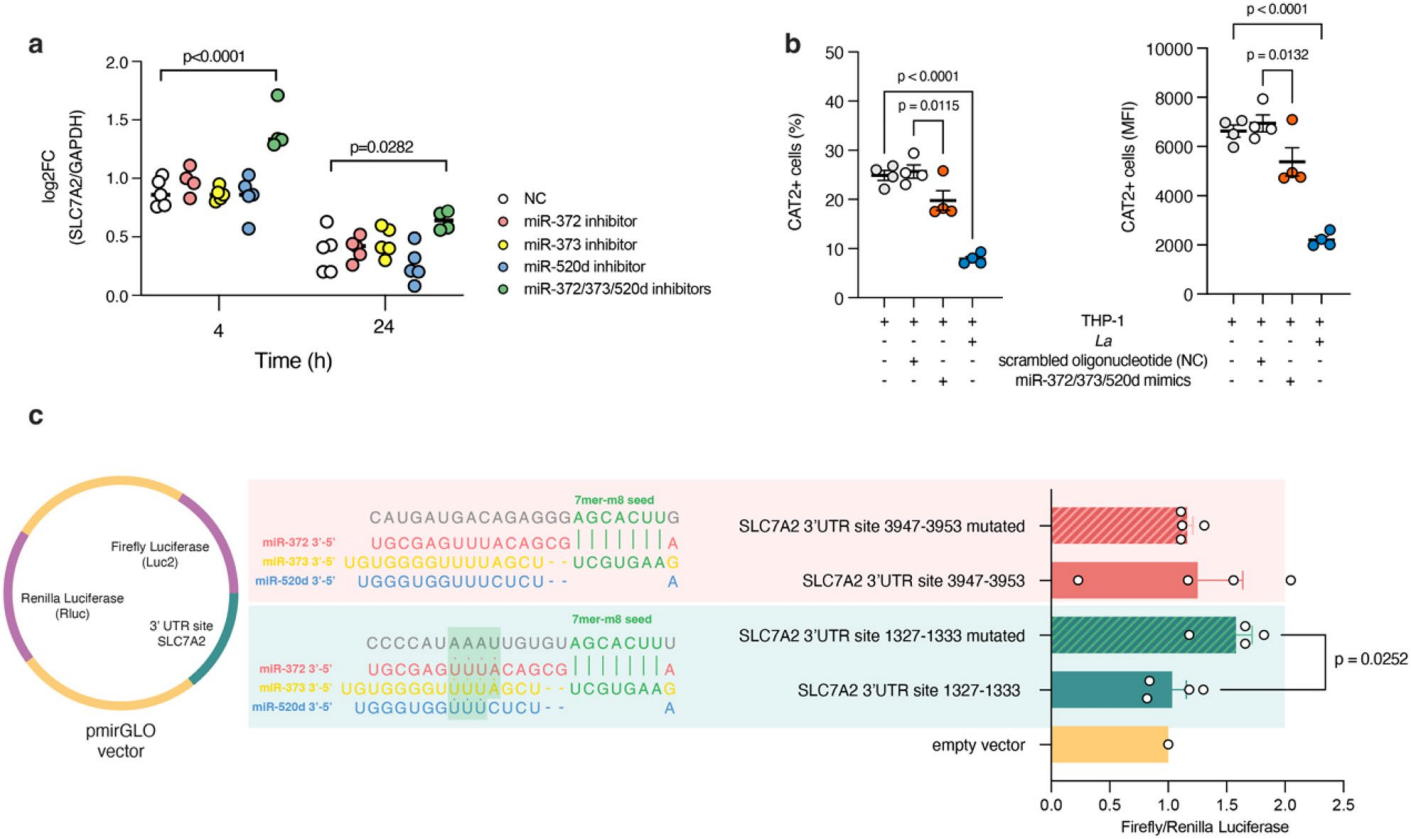
Validation of miR-372/373/520 interaction with SLC7A2/CAT2. (**a**) RT-qPCR quantification of SLC7A2 (CAT2, cationic amino acid transporter) mRNA. (**b**) Flow cytometry assessment of percentage (%) of cells expressing CAT2 and protein level of CAT2 by mean of fluorescence intensity (MFI), (**c**) relative luciferase activity (Firefly/Renilla) for CAT2 mRNA binding site. The sequences represent miRNA binding sites in the 3′UTR from the TargetScan database. NC: negative control. Error bars represent means ± sem. Significant p values (P < 0.05) are shown in the figure, and were obtained by one-sample (**a**) or two-sample (**b**,**c**) t test.

However, when quantifying mRNA levels upon miRNA inhibition, we observed an increase in SLC7A2 transcript, indicating that *Leishmania*-induced miR-372/373/520 could avoid CAT2 upregulation (Fig. 5a). In fact, after measuring CAT2 protein levels using flow cytometry, we saw that *Leishmania* infection reduced both the intensity and percentage CAT2-expressing cells compared to non-infected THP-1 (Fig. 5b). Similar effect occurred comparing miR-372/373/520 mimics-transfected macrophages compared to NC, confirming that these miRNAs could reduce CAT2 levels (Fig. 5b).

To gather additional evidence in support of a role for miR-372/373/520 in the repression of CAT2, we conducted luciferase activity assays. We co-transfected pmiRGLO vectors containing 2 distinct seed binding motifs from the SLC7A2 3′UTR, along with miR-372/373/520 mimics in RAW cells. We observed that both the standard and control mutated sequences from the putative binding site at 3947–3953 displayed similar levels of luciferase activity. In contrast, the standard sequence from the 1327–1333 putative binding site presented a lower luciferase signal compared to the control mutated sequence following transfection with miR-372/373/520 mimics, confirming a functional miRNA binding site (Fig. 5c). Interestingly, when comparing the sequence complementarity for both 3′UTR portions, we observed that only the 1327–1333 region presented conserved additional complementarity to the miRNAs, while the 3947–3953 region matched exclusively within the seed portion. This finding suggests that, despite seed sequence being a critical determinant of miRNA function, additional complementarity in nucleotides 14–17 is essential for CAT2 repression.

## Discussion

In this study, our primary objective was to examine the modulation and functional role of human miRNAs after *L. amazonensis* infection in THP-1 macrophages. Out of the 84 miRNAs examined, 8 miRNAs exhibited upregulation following infection (Fig. 1, Suppl. Table 1). Notabily, among the upregulated miRNAs were miR-372, miR-373, miR-520d, miR-302a, and miR-302c, all belonging to the same miRNA family, characterized by a shared seed sequence responsible for the complementary binding to the target mRNAs.

The miRNA family identified in our study was also found to be upregulated in THP-1 macrophages infected with other *Leishmania species*. For instance, *L. infantum*-infected THP-1 macrophages and plasma of infected patients exhibited increased levels of miR-302a (at 6 h), miR-302b, miR-372, and miR-373 (at 6 and 24 h) compared to healthy individuals^32^. Similarly, THP-1 macrophages infected with *L. braziliensis* upregulate miR-372, miR-302a, and miR-302b after 6 and 24 h and miR-520d after 24 h^24^.

Interestingly, prior studies by our research group have reported an increased expression of miR-294^22^ and miR-302d^21^ in BMDM from BALB/c mice infected with *L. amazonensis*, both sharing the same seed sequence as the human miRNA family identified herein. The miR-372–373 cluster in humans is homologous to the murine miR-294–295 cluster^44^, while the miR-302 cluster is conserved among humans and mice^45^, indicating that *Leishmania*-induced expression of miRNAs containing the AAGUGCU seed sequence may represent a conserved mechanism across host species.

The aforementioned seed sequence is present in 28 human miRNAs and has been designated an “oncomotif” due to its capacity to enhance cancer cell proliferation by interfering with the cell cycle^46^. This sequence is also associated with pluripotency and was initially described in germ and embryonic cells^47^.

Using an arginine-deficient *L. amazonensis* model, we demonstrated that both *La*-WT and *La-arg*^*−*^ present similar infection level at 4 h, but at 24 h, *La-*WT exhibited a higher infection index. Similarly, while *La-*WT upregulated miR-372, miR-373, and miR-520d at both 4 and 24 h after infection, *La-arg*^*−*^ induced their upregulation at the 4-h timepoint, failing to sustain these miRNAs until 24 h of infection. The coincidence of miRNA expression and infection index, as previously noted in our data regarding miR-294^22^, led us to propose that the human miR-372/373/520d family may be important to promote *Leishmania* infection.

We found that inhibiting miR-372 alone resulted in a reduction in infection, whereas inhibition of miR-373 or miR-520d alone did not have a significant effect. Interestingly, the collective inhibition of all three miRNAs exhibited a synergistic increase of the observed effects (Fig. 2). Since the simultaneous inhibition of miRNAs yielded effects that were approximately twice as strong as inhibiting miR-372 alone, we can infer the existence of a cooperative mechanism among miRNAs from the same family in influencing the outcome of infection.

The lack of effect when inhibiting miR-373 and miR-520d alone may be explained by potential compensatory effects among these miRNAs. This observation may also be ascribed to non-shared targets within the same miRNA family due to additional complementarity in their 3′ portions^48^. Experimental and mathematical evidence based on the thermodynamics of base pairing has shown that miRNAs from the miR-372/373/520 family exhibit varying affinities for their targets due to other determinants beyond seed^49^. Transfecting macrophages with a combination of miR-372/373/520d miRNAs mimics resulted in an increase in the proportion of infected macrophages (Suppl. Fig. 5), further supporting that these miRNAs promote infection.

Since the first years after its discovery miRNAs have been implicated in metabolism^50^. Recently, miR-372 and its mice homolog miR-294 were implicated in the deregulation of lipid metabolism when associated to the lncRNA NEAT1^51^. Whether lncRNA could mediate metabolic rewiring in our model is still an open question, since lncRNA expression during *Leishmania* infection was only recently reported^52,53^.

Because miR-372/373/520 can have multiple targets with largely diverse functions (Suppl. Table 4), metabolomics is a valuable tool for unraveling the impact of these miRNAs on cell phenotype. However, it is noteworthy that few studies have examined metabolomic changes following interference with post-transcriptional modulators, such as for the miR-155, miR-124, and miR-125b^54–56^. Despite being an important technique for elucidating the physiological status of parasites or infected macrophages, which relies on a variety of post-transcriptional and post-translational mechanisms, metabolomics techniques also remain underexplored in the study of leishmaniasis.

In this investigation, MS-based metabolomics was employed to assess the average and significance of changes in metabolite levels within non-infected and infected macrophages, with or without miRNA inhibition. We aimed to systematically investigate whether combinatorial miRNA inhibition leads to metabolic alterations that may explain how infectivity is affected by miRNA action. Our findings validated the capacity of miRNAs to modulate the metabolome, as evidenced by distinct group separation in the supervised PLS-DA model of positive mode data (Fig. 3a). Conversely, the PLS-DA models of data obtained in negative polarity failed to distinguish groups due to a low number of differentially expressed anionic analytes.

Univariate statistical analysis revealed that both *Leishmania* infection and miRNA inhibition lead to a modulation of numerous metabolites, suggesting their role in regulating metabolic pathways. We revealed a surprising dichotomic pattern regarding the urea cycle and polyamine production metabolites. While infection led to an increased the expression of ornithine, citrulline, argininosuccinate, and putrescine, inhibiting the infection-induced miRNAs resulted in a decrease in arginine, ornithine, citrulline, argininosuccinate, putrescine, and spermine levels.

These metabolomic results are consistent with previous studies highlighting the essential role of polyamines in *Leishmania* infectivity^57^. A recent study reported increased ornithine and citrulline levels, but not arginine, in patients with diffuse cutaneous leishmaniasis^58^. The increase in ornithine, citrulline, and putrescine has also been shown in *L. amazonensis* infection in BALB/c and C57BL/6 BMDMs^5,6^.

In *L. donovani*-infected THP-1 macrophages, there is an increase in putrescine and spermidine^59^, while our data indicate that *L. amazonensis* increases putrescine only (Fig. 3). Another noteworthy factor when comparing the two models is that the study with *L. donovani* demonstrated that siRNA or chemical inhibition of CAT2 reduces arginine uptake, polyamine production, and survival of the parasite in THP-1 macrophages^59^. In contrast, in our study, the miRNAs induced by *L. amazonensis* target CAT2 (Fig. 5), while there is an increase in polyamine production (Fig. 3). In addition to the difference in the studied species and the pathophysiology of the diseases induced by both species, the *L. donovani* study focused on 48 h post-infection in vitro, while our study focused on 4 and 24 h post-infection.

The observed increase in ornithine, argininosuccinate, and citrulline during infection aligns with the metabolic profile of M2-polarized human macrophages^60^. Since NOS2 is not active in human macrophages^15,16^, the increase in citrulline levels in M2 macrophages can be explained by the activity of OTC^60^. OTC is part of the urea cycle and is a putative target of the miR-372/373/520d family (Table 3). On the other hand, we observed a reduction in citrulline levels upon inhibition of miR-372/373/520d (Fig. 3). It has recently been shown that M1 macrophages in humans and mice decrease citrulline levels for pro-inflammatory activation^61^. Furthermore, citrulline can lead to ornithine production via the laccase domain-containing 1 enzyme in murine macrophages^62^.

Interestingly, miRNA inhibition caused a strong increase in N-acetylspermidine, which was significantly reduced by *Leishmania* infection (Fig. 3). This metabolite can be assumed to be derived from the host since *Leishmania* lacks the enzyme spermidine/spermine N1-acetyltransferase (SSAT)^63^. Acetyl spermidine is destined for degradation or export^64,65^ and is an important component controlling the levels of polyamines in cells. In viral infections, type I interferons induce SSAT activity, leading to polyamine acetylation and decreased replication of viral particles^66^.

Polyamines play contradictory roles in maintaining cellular oxidative homeostasis. They protect against free radical-mediated damage by promoting glutathione production, but they can also generate reactive oxygen species (ROS) through polyamine oxidase (PAOX), which catalyzes the oxidation of N-acetyl spermidine to putrescine^65^. ROS production via PAOX is regulated by miR-124 and is implicated in the development of *Helicobacter pylori*-associated gastric cancer^67^.

This phenotypic shift not only affects macrophage response to infection, but may also affect metabolism in the parasite itself. In this way, we found reduced trypanothione levels, although this change seems to be an indirect effect of reduced glutathione levels (Suppl. Table 3), which is a host substrate required for trypanothione synthesis^68^.

Considering that miRNAs from the same family can inhibit similarly predicted targets, we investigated the enrichment of target genes of the miR-372/373/520 family using the TargetScan database, along with the altered metabolites upon inhibition of these miRNAs in infected macrophages (Table 3). Among enriched pathways, the urea cycle and polyamine synthesis belong to arginine metabolism, corroborating transcriptomic studies conducted on C57BL/6 mouse peritoneal macrophages, which demonstrated, among other pathways, enrichment of arginine metabolism after 4 h of *L. major* infection^69^.

The miR-372/373/520 family putatively targets numerous urea cycle-related transcripts, notably encompassing nuclear-encoded mitochondrial transporters and enzymes. These include the ornithine/citrulline antiporter ORC1 (encoded by the SLC25A15 gene), arginase 2 (ARG2), OTC, and the N-acetylglutamate synthetase (NAGS). The miR-372/373/520 also targets the cytosolic enzyme ASL (Table 3). Among putative targets related to polyamine metabolism, there are enzymes directly or indirectly involved in polyamine biosynthesis, such as the adenosylmethionine decarboxylase 1 (AMD1), polyamine oxidase (PAOX), agmatinase (AGMAT) and the antizyme inhibitor 1 (AZIN1) (Table 3).

Conversely, all the above-mentioned enzymes mediate reactions producing metabolites that are reduced upon miRNA inhibition (Suppl. Fig. 8). Since miRNAs act mostly in target repression it is still unclear why inhibiting miRNAs would lead to diminished metabolite uptake or enzyme activity. This indicates that indirect and unidentified targets could contribute to the observed phenotype.

Several known metabolite transporters are putative targets of the miR-372/373/520 family, among those the CAT2, the main arginine transporter in activated macrophages^13,14^. Similar to the infectivity assays, the inhibition of a single miRNA did not show regulation at the mRNA level, but the combined inhibition of miR-372, miR-373, and miR-520d led to positive regulation of the SLC7A2 mRNA (Fig. 5). Although some miRNAs can directly affect protein levels without changing mRNA levels, miRNAs that affect mRNA typically influence protein levels^70^. Here, transfection of miR-372/373/520 mimics or *Leishmania* infection reduced CAT2 protein expression (Fig. 5), suggesting a role for *Leishmania*-induced miRNAs in CAT2 regulation.

The validation of miRNA binding sites within SLC7A2 gene by miR-372/373/520 was conducted through luciferase-based assays, considering the potential impact of the context of the 3′ UTR on miRNA affinity, as influenced by sequences adjacent to the seed site^71^. Our results indicated that the presence of the 1327–1333 position site in the SLC7A2 UTR was crucial for miRNA-mediated regulation (Fig. 5). Mutation of the seed sequence in this region abrogated the inhibitory effect of the miRNA mimics on luciferase activity, thus confirming that miR-372, miR-373, and miR-520d directly target the 3′ UTR of SLC7A2 mRNA to regulate CAT2 expression. However, the seed in position 3947–3953 was not functional, likely attributed to additional pairing in the 3′ portions, consistent with existing literature suggesting an important role of additional 3′ end pairing in enhancing target repression and determining specificity of paralog miRNAs^72^.

It is important to note that miRNA-mediated regulation is a complex process and can involve multiple targets and pathways. While our study focused on validating the regulation of CAT2 by miR-372, miR-373, and miR-520d, other miRNAs and target genes may also be involved in the macrophage response to *Leishmania* infection. Further studies are needed to elucidate the broader regulatory network and the precise mechanisms by which miRNAs modulate macrophage functions during *Leishmania* infection.

In summary, we found that infection-induced polyamine production depends on miR-372/373/520 availability, as evidenced by the reduction of arginine levels and its downstream metabolites of both the urea cycle and polyamine production upon miRNA inhibition. This highlights the miR-372/373/520 family as a key modulator of macrophage by favoring *Leishmania* survival through *Leishmania*-induced metabolic reprogramming.

Understanding the intricate role of miRNAs in host-parasite interactions provides valuable insights into the molecular mechanisms underlying the pathogenesis of leishmaniasis. Notably, miRNAs emerge as promising host-directed therapeutic targets.

### Limitations

Since miRNAs have multiple targets, selective chemicals or siRNA could unravel individual enzymes' contributions to the immunometabolic response during *Leishmania* infection.

### Supplementary Information

Supplementary Information.

Supplementary Table 4.

## Data Availability

The miRNA RT-qPCR array data are available on the Gene Expression Omnibus (GEO) platform under the accession number GSE242513 (https://www.ncbi.nlm.nih.gov/geo/query/acc.cgi?acc=GSE242513). Other raw datasets used and/or analyzed during the current study are available from the corresponding author upon reasonable request.
