# Supplementary material for: Early Leishmania infectivity depends on miR-372/373/520d family-mediated reprogramming of polyamines metabolism in THP-1-derived macrophages
Source: Sci Rep. 2024 Jan 10;14:996. doi: 10.1038/s41598-024-51511-y (PMC10781704; doi:10.1038/s41598-024-51511-y)
Supplement: Supplementary file 1 — Supplementary Information. [file 41598_2024_51511_MOESM1_ESM.docx]

**Supplementary material**

**Early *Leishmania* infectivity depends on miR-372/373/520d family-mediated reprogramming of polyamines metabolism in THP-1-derived macrophages**

Fernandes, J.C.R.^1,2^; Muxel, S.M.^3^; López-Gonzálvez, M.A.^4^; Barbas, C.^4^; Floeter-Winter, L.M^2*^

1 Instituto de Medicina Tropical da Faculdade de Medicina da Universidade de São Paulo (IMT-FMUSP), São Paulo, Brazil

2 Instituto de Biociências da Universidade de São Paulo (IB-USP), São Paulo, Brazil

3 Instituto de Ciências Biomédicas da Universidade de São Paulo (ICB-USP), São Paulo, Brazil

4 Centre for Metabolomics and Bioanalysis (CEMBIO), Department of Chemistry and Biochemistry, Facultad de Farmacia, Universidad San Pablo-CEU, CEU Universities, Urbanización Montepríncipe, Boadilla del Monte, 28660 Madrid, Spain.

* corresponding author: lucile@ib.usp.br


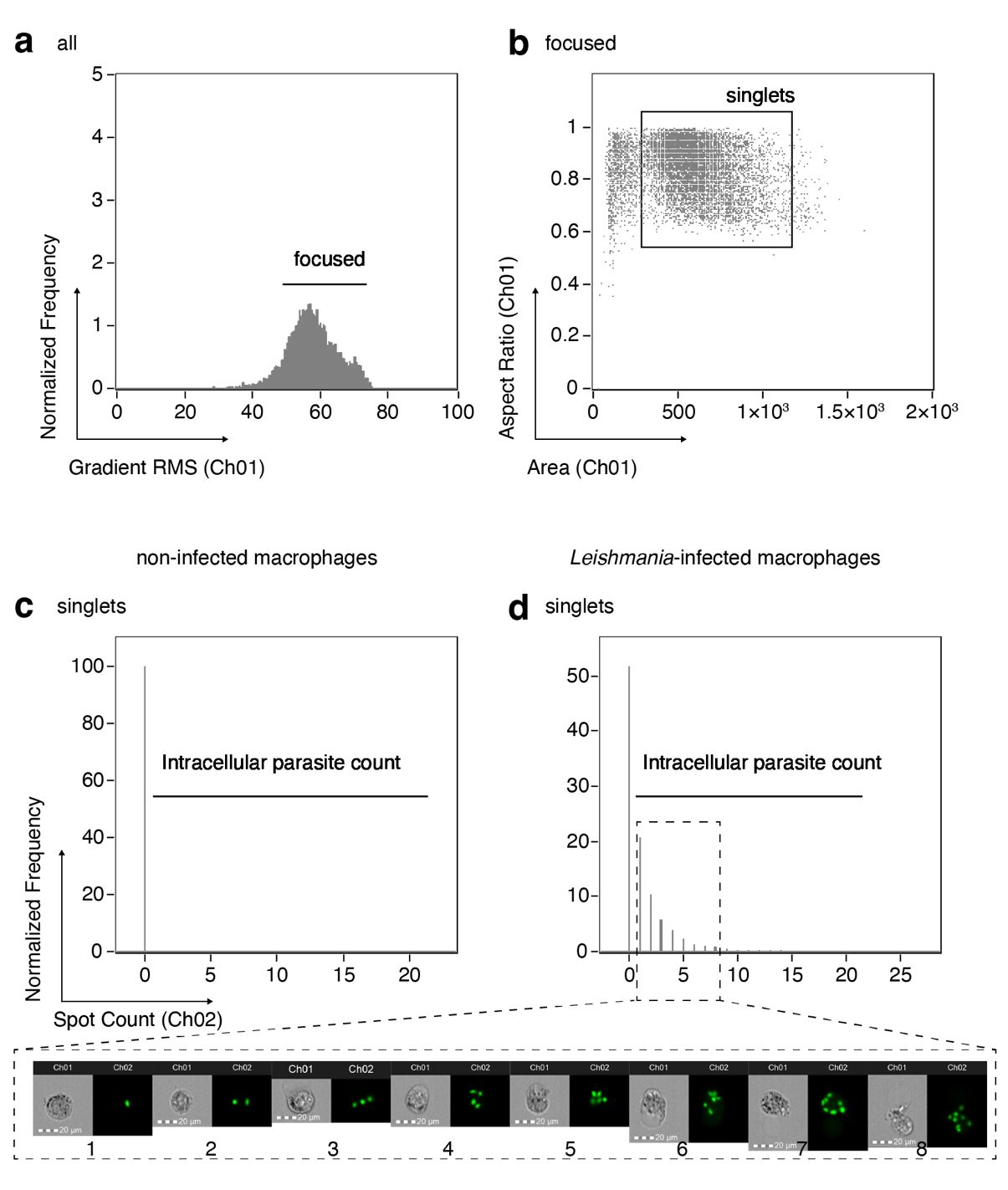


Supplementary Figure 1. Imaging flow cytometry gating strategy for the analysis of *L. amazonensis* infection in THP-1-derived macrophages. a, gate for focused images, b, gate for single cells, c and d, gate for calculation of the frequency of infected cells and intracellular parasite count, with image examples.

**
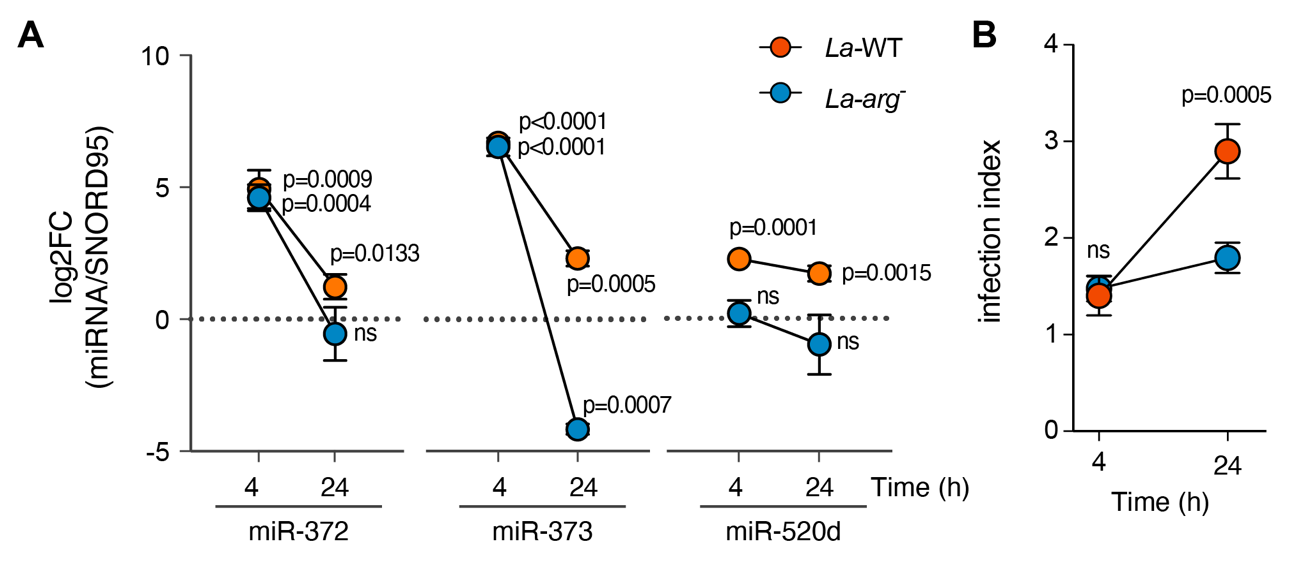
**

Supplementary Figure 2. Expression of the miRNA-372/373/520d family and infectivity of wildtype *La* (La-WT) or arginase-knockout *La* (*La-arg^-^*) in THP-1 macrophages. a, RT-qPCR of miR-372, miR-373, and miR-520d expression; b, infection index obtained through imaging flow cytometry (percentage of infected macrophages × amastigote per macrophages). Error bars represent ± s.e.m. P values are indicated in the figure and were obtained by one sample t test (N=3-4).


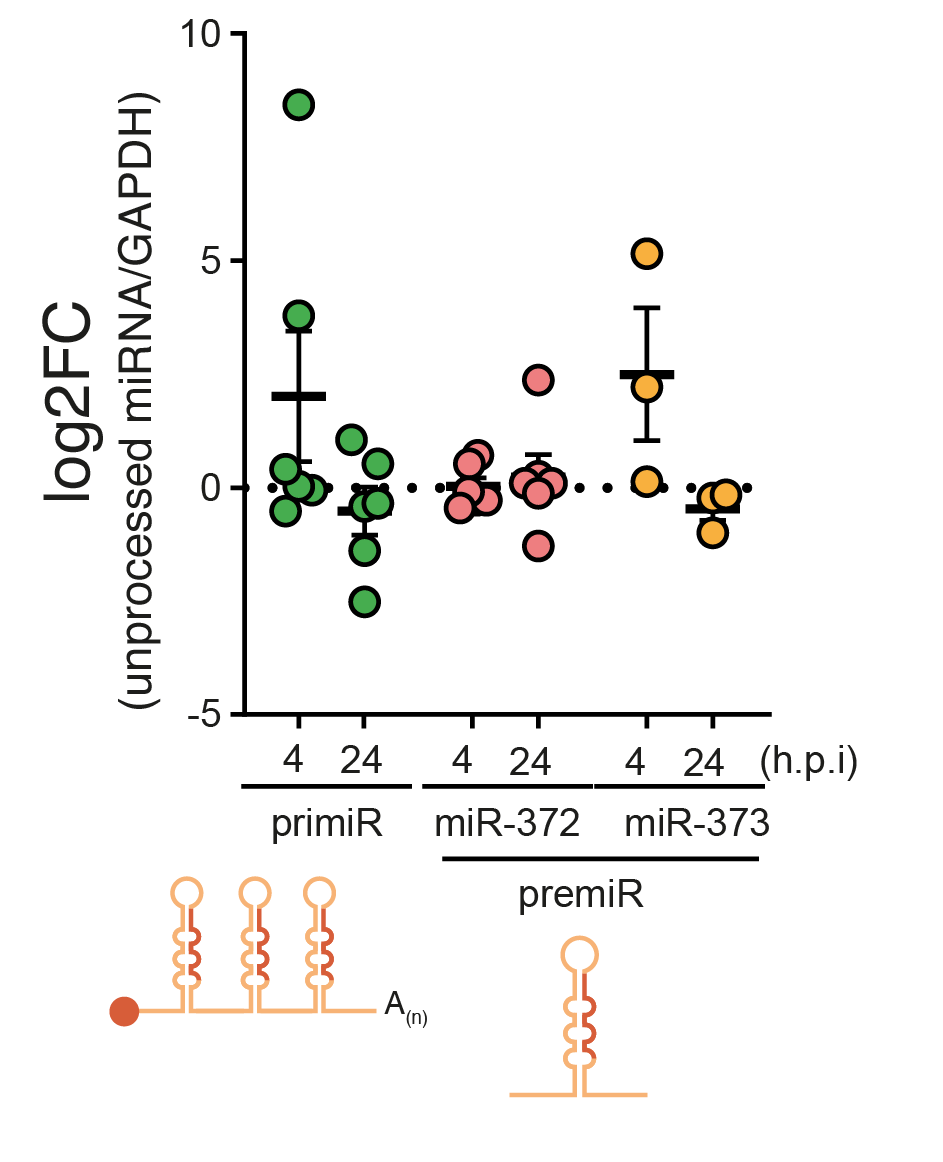


Supplementary Figure 3. Biogenesis intermediates of pri- and premiR-372 and 373. Pre and pri miRNA levels were quantified by RT-qPCR. Error bars represent ± s.e.m. P values are indicated in the figure and were obtained by one sample t test (N=3-6).


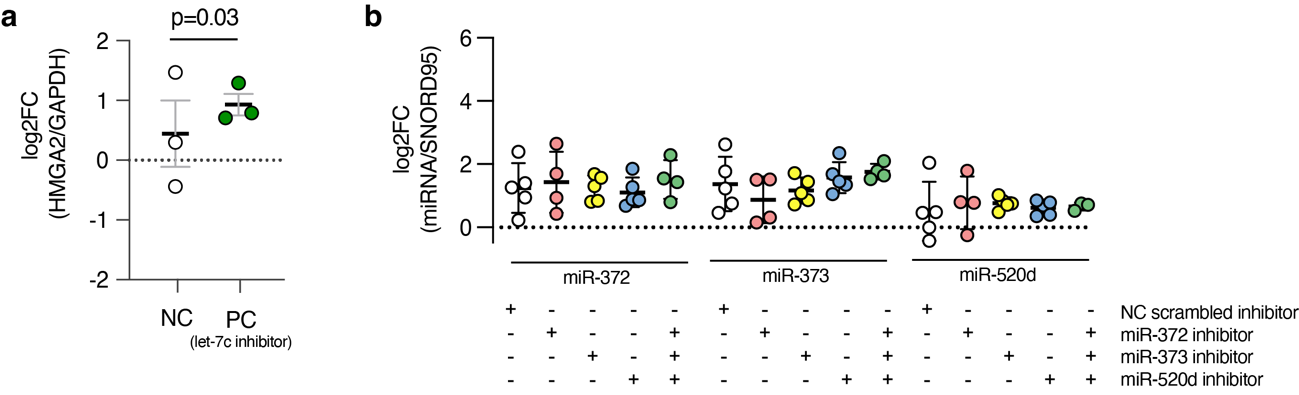


Supplementary Figure 4. Controls for miRNA inhibition assay by RT-qPCR. a, Validation of transfection protocol by quantification of HMGA2 mRNA level as a known target for let-7c, used as commercial positive control and b, quantification of miR-373, miR-372, and miR-520d miRNAs upon each miRNA inhibition alone and miRNA inhibition in combination. Error bars represent log2FC values ± s.e.m. P values are indicated in the figure when significant (P<0.05) and were obtained by one sample t test.


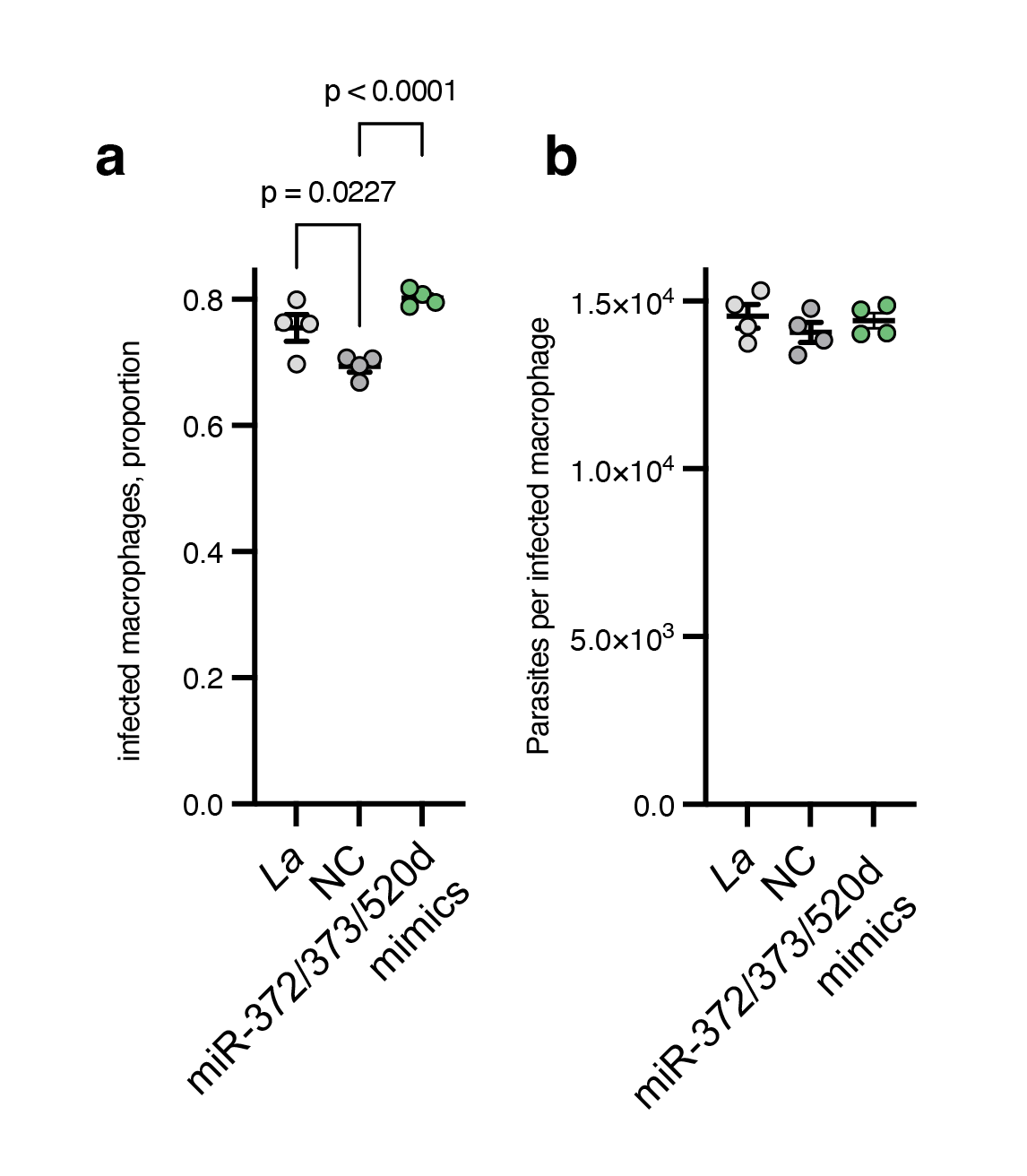


Supplementary Figure 5. MiRNA miR-372/373/520d mimics increase *Leishmania amazonensis* infectivity in THP-1 macrophages. a, Proportion of infected macrophages. b, Parasites per infected macrophage. Error bars represent means ± s.e.m. P values were assessed by ANOVA Dunnet’s posthoc test and P < 0.05 are depicted in the figure.


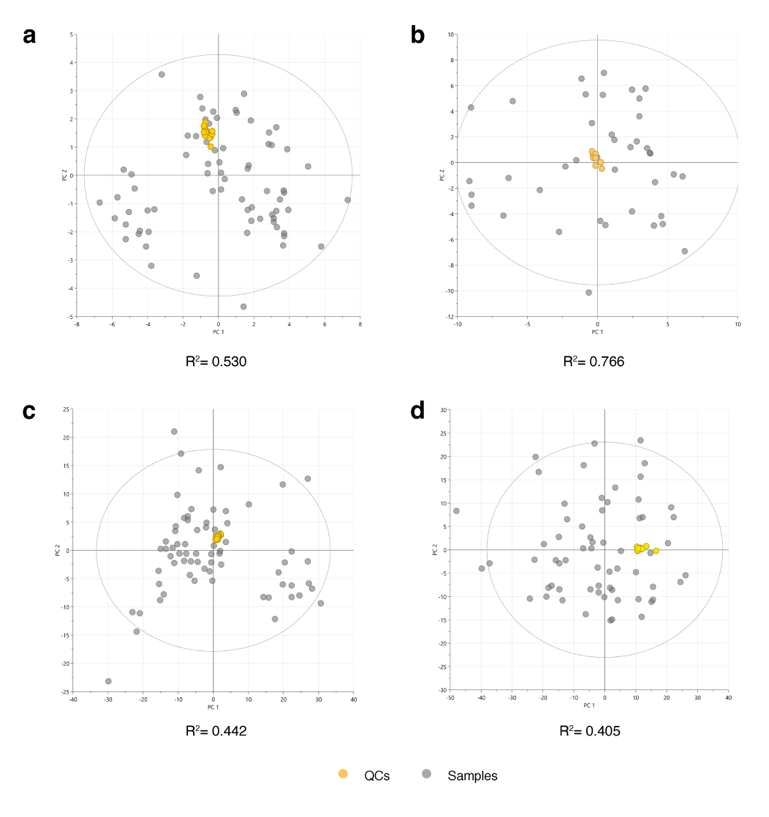


Supplementary Figure 6. Quality control by unsupervised PCA-X highlighting QC samples distribution. a, CE-ESI(+)-TOFMS, b, CE-ESI(-)-TOFMS, c, HILIC-LC-(+)-QTOF and d, HILIC-LC-(-)-QTOF. QC: Quality control.


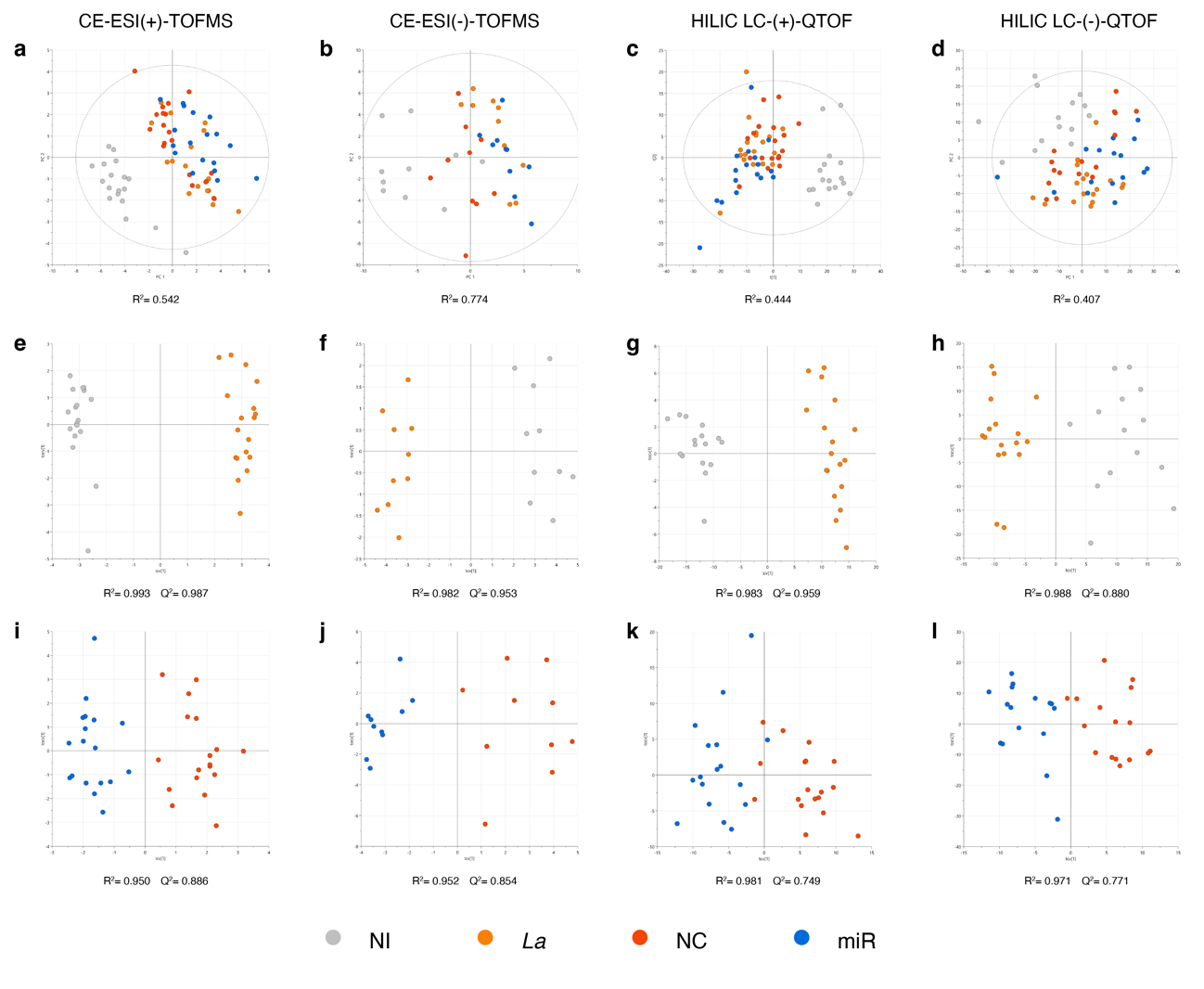


Supplementary Figure 7. Multivariate data analysis of multiplatform metabolomics data. Right columns depict data from positive (+) and negative (-) mode capillary electrophoresis-mass spectrometry (CE-MS) and left columns from positive (+) (N=17-18) and negative (-) (N=10) mode HILIC-LC-QTOF (N=14-17). a-d, PCA-X; e-h, OPLS-DA for *La* × NI comparison and i-l, OPLS-DA for miR × NC comparison. NI: non-infected macrophage, *La*: *L. amazonensis*-infected macrophage, NC: *L. amazonensis*-infected macrophage treated with negative control and miR: *L. amazonensis*-infected macrophage treated with miR-372/373/520d inhibitors.


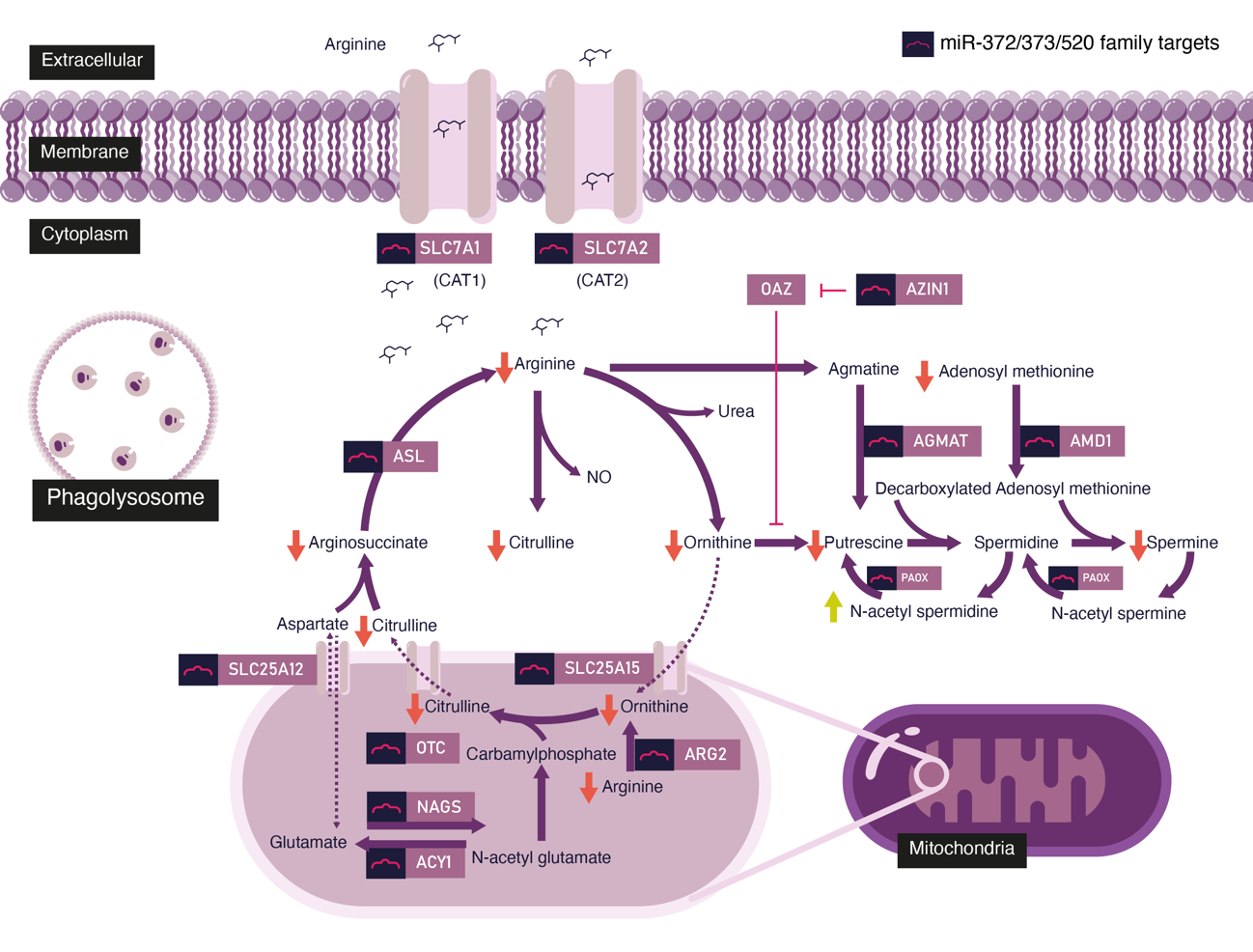


Supplementary Figure 8. miR-302/372/373/520 family targets multiple transcripts encoding arginine metabolism-related proteins and are essential for polyamine and trypanothione production. ACY1: aminoacylase 1, AGMAT: agmatinase, AMD1: S-adenosylmethionine decarboxylase, ARG2: arginase 1, ASL: argininosuccinate lyase, AZIN1: antizyme inhibitor 1, NAGS: N-acetylglutamate synthase, OTC: ornithine transcarbamylase, PAOX: peroxisomal N1-acetyl-spermine/spermidine oxidase, SLC25A12: solute carrier family 25 member 12, SLC25A15: solute carrier family 25 member 15, SLC7A1: solute carrier family 7 member 1 and SLC7A2: solute carrier family 7 member 2.


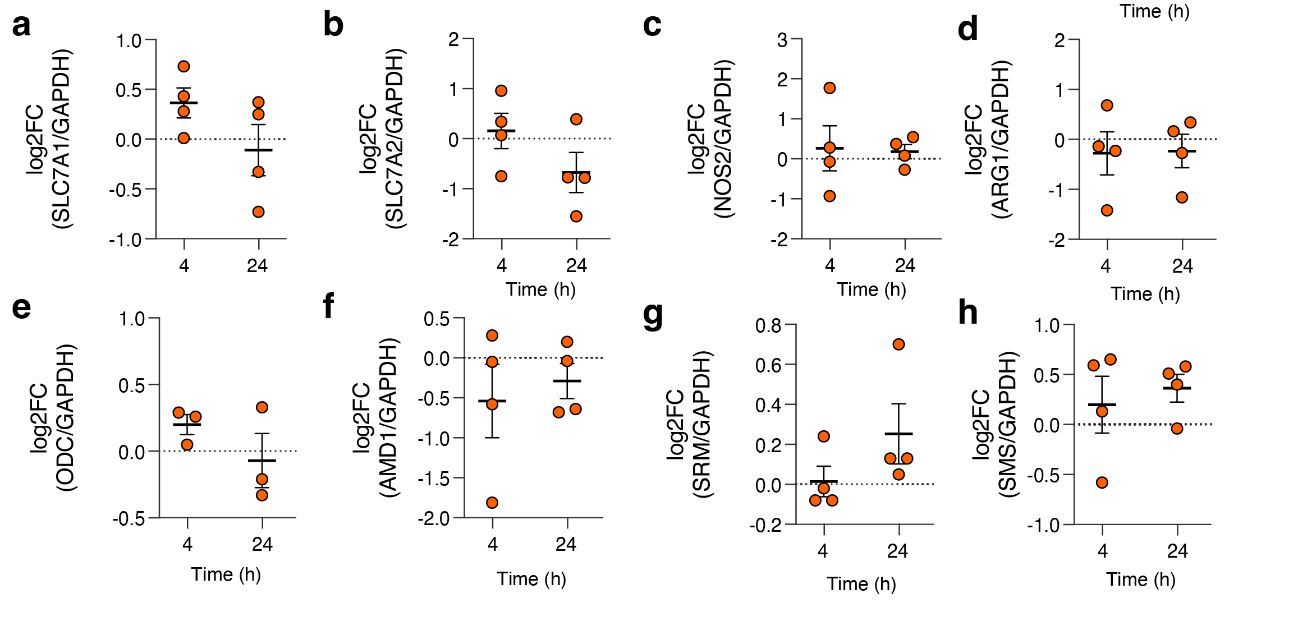


Supplementary Figure 9. Quantification of mRNA levels of enzymes related to arginine metabolism. Error bars represent log2FC values ± s.e.m. P values are indicated in the figure when significant (P<0.05) and were obtained by one sample t test. SLC7A1: solute carrier transporter 7A member 1, SLC7A2: solute carrier transporter 7A member 2, NOS2: nitric oxide synthase 1, ARG1: arginase 1, ODC: ornithine descarboxylase, AMD1: adenosyl methionine decarboxilase, SRM: spermidine synthase, and SMS: spermine synthase.

***Supplementary tables***

Supplementary Table 1. Expression of 84 miRNAs related to immune response quantified after 4 and 24 hours of infection of *L. amazonensis* in THP-1-derived macrophages. Values of -1< Log2FC > 1 were submitted to statistical analysis. P values were obtained from 2-sample, two-tailed Student’s t test comparing infected to non-infected macrophages (P) and were corrected (P*adj*) by the two-stage linear method of Benjamini, Krieger and Yekutiele. P*adj* < 0.05 was considered statiscally significant.

| *4h*  Log2FC (P; Padj) | *24h*  Log2FC (P; Padj) |  |
| --- | --- | --- |
| hsa-let-7a-5p | -0.4141 | -0.5888 |
| hsa-let-7b-5p | 0.07025 | 0.46069 |
| hsa-let-7c-5p | -0.0666 | -0.1201 |
| hsa-let-7d-5p | 0.29184 | 0.29772 |
| hsa-let-7e-5p | 0.42191 | 0.03844 |
| hsa-let-7f-5p | -0.0249 | 0.00101 |
| hsa-let-7g-5p | 0.01207 | 0.19535 |
| hsa-let-7i-5p | -0.6652 | 0.40795 |
| hsa-miR-101-3p | 0.00346 | 0.49313 |
| hsa-miR-106b-5p | **-1.5513**  **(0.357094; 0.102259)** | 0.40577 |
| hsa-miR-125a-5p | 0.18625 | -0.1494 |
| hsa-miR-125b-5p | 0.3575 | -0.0395 |
| hsa-miR-128-3p | 0.12948 | 0.14418 |
| hsa-miR-130a-3p | 0.19131 | -0.4229 |
| hsa-miR-130b-3p | -0.3432 | 0.0827 |
| hsa-miR-1324 | 0.68715 | 0.59579 |
| hsa-miR-144-3p | -0.3553 | -0.1076 |
| hsa-miR-145-5p | -0.2231 | 0.05492 |
| hsa-miR-15a-5p | 0.08366 | 0.14574 |
| hsa-miR-15b-5p | -0.0274 | 0.11143 |
| hsa-miR-16-5p | -0.1504 | 0.25036 |
| hsa-miR-17-5p | 0.38626 | 0.26123 |
| hsa-miR-181a-5p | 0.29842 | 0.33697 |
| hsa-miR-181b-5p | 0.13435 | -0.1912 |
| hsa-miR-181c-5p | -0.391 | 0.57114 |
| hsa-miR-181d-5p | 0.23756 | -0.0577 |
| hsa-miR-186-5p | -0.0583 | 0.51096 |
| hsa-miR-195-5p | -0.1644 | 0.1564 |
| hsa-miR-19a-3p | 0.7946 | 0.54122 |
| hsa-miR-19b-3p | 0.58756 | 0.3364 |
| hsa-miR-202-3p | **3.36772**  **(0.001002;**  **0.001590)** | 0.70699 |
| hsa-miR-20a-5p | 0.07341 | 0.39298 |
| hsa-miR-20b-5p | 0.2806 | 0.41251 |
| hsa-miR-21-5p | 0.18396 | 0.04768 |
| hsa-miR-211-5p | **1.17498**  **(0.183440; 0.057784)** | 0.65682 |
| hsa-miR-23a-3p | 0.06392 | -0.4199 |
| hsa-miR-23b-3p | -0.2428 | -0.123 |
| hsa-miR-29a-3p | 0.30498 | 0.35907 |
| hsa-miR-29b-3p | -0.1407 | 0.22959 |
| hsa-miR-29c-3p | 0.36255 | 0.22589 |
| hsa-miR-300 | **1.35586**  **(0.000140; 0.000147)** | -0.3644 |
| hsa-miR-301a-3p | 0.42557 | -0.4925 |
| hsa-miR-301b-3p | 0.42632 | 0.06323 |
| hsa-miR-302a-3p | 0.75719 | 0.43093 |
| hsa-miR-302b-3p | 0.39375 | -0.6565 |
| hsa-miR-302c-3p | **1.27435**  **(0.020283;**  **0.057950)** | -0.4383 |
| hsa-miR-30a-5p | -0.3281 | 0.11023 |
| hsa-miR-30b-5p | -0.1769 | -0.7406 |
| hsa-miR-30c-5p | -0.3103 | -0.0569 |
| hsa-miR-30d-5p | -0.122 | 0.52677 |
| hsa-miR-30e-5p | 0.05728 | 0.52717 |
| hsa-miR-340-5p | 0.85758 | 0.54468 |
| hsa-miR-34a-5p | -0.0345 | 0.4917 |
| hsa-miR-34c-5p | -0.0032 | 0.38869 |
| hsa-miR-372-3p | **4.89975**  **(<0.0001; <0.0001)** | **1.58275**  **(0.04524; 0.09500)** |
| hsa-miR-373-3p | **5.8643**  **(0.025550; 0.010257)** | **1.65787**  **(0.16681; 0.17515)** |
| hsa-miR-374a-5p | 0.10769 | 0.13093 |
| hsa-miR-381-3p | **2.83015**  **(0.000018; 0.000028)** | 0.21325 |
| hsa-miR-410-3p | 0.79751 | -0.5291 |
| hsa-miR-424-5p | 0.35851 | 0.36983 |
| hsa-miR-449a | -0.1308 | 0.34028 |
| hsa-miR-449b-5p | 0.10528 | 0.1564 |
| hsa-miR-454-3p | -0.2812 | -0.3425 |
| hsa-miR-497-5p | -0.093 | -0.0821 |
| hsa-miR-511-5p | 0.48512 | -0.0547 |
| hsa-miR-513b-5p | -0.3827 | -0.4614 |
| hsa-miR-519c-3p | -0.236 | -0.5888 |
| hsa-miR-519d-3p | 0.56013 | -0.2711 |
| hsa-miR-520d-3p | **3.01403**  **(0.010257;**  **0.026050)** | 0.4321 |
| hsa-miR-520e | 0.35997 | 0.00518 |
| hsa-miR-524-5p | 0.58919 | -0.2292 |
| hsa-miR-543 | **2.97089**  **(0.000969;**  **0.001230)** | -0.3432 |
| hsa-miR-545-3p | **1.94703**  **(0.007140;**  **0.013600)** | 0.0686 |
| hsa-miR-548c-3p | -0.2634 | 0.31034 |
| hsa-miR-548d-3p | -0.4434 | 0.40479 |
| hsa-miR-548e-3p | 0.48553 | 0.30147 |
| hsa-miR-590-5p | 0.4351 | 0.29772 |
| hsa-miR-607 | -0.5031 | -0.3313 |
| hsa-miR-655-3p | -0.1581 | 0.0049 |
| hsa-miR-656-3p | 0.31174 | -0.7269 |
| hsa-miR-875-3p | 0.31615 | -0.4465 |
| hsa-miR-9-5p | 0.08488 | 0.04222 |
| hsa-miR-93-5p | 0.33971 | -0.1589 |
| hsa-miR-98-5p | -0.0233 | 0.00518 |

Supplementary Table 2. Annotation of metabolites determined as significant (p FDR < 0.05) in the data set of *La*-infected THP-1 versus non-infected THP-1, referred to as *La* × NI.; RSD, relative standard deviation; FC, fold change; FDR, false discovery rate; P(corr), partial correlation coefficient; VIP, variable importance in projection.

| Name | Mass | RSD | Log2FC | p FDR | P(corr) | VIP |
| --- | --- | --- | --- | --- | --- | --- |
| Glycine | 75.0325 | 3.55 | -0.79 | 9.4×10^-5^ | -0.69 | 1.04 |
| Putrescine | 88.0995 | 7.52 | 3.36 | 3.5×10^-11^ | 0.98 | 2.18 |
| Alanine | 89.0479 | 2.91 | 1.71 | 1.3×10^-8^ | 0.91 | 1.54 |
| Lactic acid | 90.0316 | 4.64 | -0.60 | 8.8×10^-7^ | -0.84 | 1.68 |
| 2-Aminobutyric acid/  3-Aminobutyric acid/  4-Aminobutyric acid | 103.0634 | 3.11 | -1.39 | 2.9×10^-5^ | -0.76 | 1.58 |
| Choline | 104.1070 | 2.79 | -1.03 | 4.3×10^-6^ | -0.86 | 1.20 |
| Serine | 105.0424 | 4.17 | -0.30 | 1.3×10^-2^ | -0.42 | 0.66 |
| Creatinine | 113.0584 | 5.49 | -1.37 | 1.5×10^-5^ | -0.78 | 1.40 |
| Maleamate | 115.0266 | 5.09 | -1.77 | 2.9×10^-5^ | -0.77 | 1.59 |
| Proline | 115.0633 | 4.19 | -0.57 | 1.1×10^-2^ | -0.50 | 1.25 |
| Succinic acid | 118.0266 | 6.13 | -0.69 | 2.6×10^-4^ | -0.79 | 1.18 |
| Niacinamide | 122.0479 | 3.14 | -0.57 | 2.3×10^-6^ | -0.75 | 1.27 |
| Taurine | 125.0141 | 28.03 | -1.53 | 1.5×10^-5^ | -0.81 | 1.36 |
| Pyroglutamic acid | 129.0428 | 4.81 | -0.83 | 3.2×10^-4^ | -0.64 | 1.01 |
| 1-Pyrroline-3-hydroxy-5-carboxylate | 129.0426 | 2.98 | -0.77 | 8.5×10^-4^ | -0.65 | 1.33 |
| trans-4-hydroxyproline | 131.0584 | 3.19 | -0.49 | 1.1×10^-2^ | -0.39 | 0.80 |
| Creatine | 131.0693 | 3.02 | -1.55 | 5.5×10^-9^ | -0.90 | 1.47 |
| Isoleucine/Leucine | 131.0943 | 3.05 | -0.30 | 2.5×10^-2^ | -0.38 | 0.71 |
| Leucine | 131.0946 | 3.74 | -0.87 | 1.6×10^-4^ | -0.71 | 1.50 |
| Asparagine | 132.0532 | 3.68 | -0.83 | 5.5×10^-5^ | -0.69 | 1.06 |
| Ornithine | 132.0900 | 3.87 | 1.91 | 5.1×10^-9^ | 0.94 | 1.63 |
| Aspartic acid | 133.0371 | 3.44 | -0.49 | 1.0×10^-3^ | -0.52 | 0.85 |
| Malic acid | 134.0213 | 6.45 | -1.35 | 2.9×10^-5^ | -0.76 | 1.57 |
| Ethanolamine phosphate | 141.0190 | 12.51 | -1.85 | 2.4×10^-6^ | -0.87 | 1.61 |
| 4-Guanidinobutyric acid | 145.0848 | 4.98 | -1.42 | 1.2×10^-8^ | -0.87 | 1.41 |
| Glutamine | 146.0684 | 5.32 | 0.42 | 7.1×10^-3^ | 0.49 | 0.75 |
| Glutamic acid | 147.0528 | 3.19 | -0.87 | 2.9×10^-5^ | -0.74 | 1.10 |
| Thiomorpholine 3- carboxylate | 147.0350 | 4.06 | -0.86 | 2.2×10^-2^ | -0.47 | 1.06 |
| Xanthine | 152.0333 | 13.95 | 2.03 | 1.6×10^-4^ | 0.67 | 1.40 |
| Histidine | 155.0691 | 4.74 | 0.77 | 1.1×10^-5^ | 0.71 | 1.01 |
| 2-Aminoadipic acid | 161.0684 | 10.15 | -2.09 | 1.1×10^-7^ | -0.85 | 1.68 |
| Carnitine | 162.1122 | 3.49 | -1.30 | 3.1×10^-8^ | -0.87 | 1.32 |
| Aconitic acid | 174.0158 | 2.60 | -1.77 | 2.8×10^-5^ | -0.76 | 1.56 |
| Citrulline | 175.0954 | 18.81 | 1.77 | 9.7×10^-6^ | 0.77 | 1.45 |
| Phosphocholine | 183.0655 | 5.25 | -1.42 | 4.6×10^-6^ | -0.86 | 1.42 |
| 2-phosphoglyceric acid/  3-phosphoglyceric acid | 185.9948 | 17.95 | -0.85 | 2.4×10^-4^ | -0.80 | 1.18 |
| N1-Acetylspermidine | 187.1677 | 6.15 | -0.70 | 1.1×10^-5^ | -0.70 | 0.96 |
| N-acetyl-glutamate | 189.0639 | 2.68 | -1.75 | 3.6×10^-5^ | -0.68 | 1.44 |
| Citric acid/Isocitric acid | 192.0277 | 5.51 | -1.47 | 4.3×10^-14^ | -0.92 | 1.44 |
| NG,NG-dimethyl-arginine | 202.1428 | 3.77 | -0.94 | 1.6×10^-5^ | -0.82 | 1.42 |
| Acetylcarnitine | 203.1153 | 6.05 | -1.82 | 2.3×10^-6^ | -0.93 | 1.61 |
| Tryptophan | 204.0873 | 8.48 | -0.51 | 4.1×10^-2^ | -0.41 | 0.96 |
| Kynurenine | 208.0838 | 25.76 | -2.01 | 4.3×10^-5^ | -0.73 | 1.71 |
| Propionylcarnitine | 217.1311 | 4.58 | -1.58 | 5.9×10^-9^ | -0.80 | 1.49 |
| Pantothenate | 219.1101 | 24.42 | -1.76 | 5.1×10^-9^ | -0.81 | 1.58 |
| Cystathionine | 222.0728 | 4.64 | ↑ ^a^ |  | 0.05 | 0.41 |
| Ribulose-5-Phosphate | 230.0188 | 4.69 | -0.66 | 2.3×10^-2^ | -0.57 | 0.94 |
| Cytidine | 243.0872 | 4.68 | -1.13 | 6.6×10^-4^ | -0.47 | 1.04 |
| Isovalerylcarnitine | 245.1610 | 3.56 | -0.95 | 4.6×10^-2^ | -0.43 | 0.96 |
| Validamine 7-phosphate | 257.0670 | 17.70 | -1.40 | 6.5×10^-4^ | -0.55 | 1.23 |
| Glycerophosphocholine | 257.1026 | 3.56 | -1.60 | 2.2×10^-9^ | -0.90 | 1.50 |
| Inosine | 268.0804 | 22.06 | -1.59 | 1.3×10^-4^ | -0.67 | 1.64 |
| Cyclic argininosuccinic acid derivative 1 | 272.1130 | 3.87 | 3.26 | 3.6×10^-8^ | 0.95 | 2.14 |
| Sedoheptulose-7-Phosphate | 290.0385 | 4.52 | -1.44 | 2.6×10^-4^ | -0.84 | 1.24 |
| 5'-Methylthioadenosine | 297.0882 | 3.26 | -1.30 | 2.9×10^-5^ | -0.82 | 1.67 |
| Glutathione | 307.0831 | 15.60 | -2.28 | 5.3×10^-4^ | -0.83 | 1.23 |
| N-Acetylneuraminate | 309.1057 | 6.52 | -2.38 | 7.6×10^-6^ | -0.83 | 1.82 |
| Beta-Citryl-glutamic acid | 321.0696 | 2.57 | -1.62 | 2.9×10^-5^ | -0.83 | 1.71 |
| UMP | 324.0359 | 2.93 | -0.76 | 4.9×10^-3^ | -0.58 | 1.30 |
| Fructose-1,6-bisphosphate | 339.9915 | 8.21 | -0.44 | 3.5×10^-2^ | -0.52 | 0.84 |
| AMP | 347.0630 | 2.11 | -0.68 | 9.5×10^-9^ | -0.82 | 1.36 |
| S-Adenosylmethionine | 398.1379 | 4.08 | -1.25 | 1.2×10^-3^ | -0.59 | 1.26 |
| UDP | 404.0020 | 2.62 | -1.06 | 2.9×10^-5^ | -0.76 | 1.58 |
| S-(2,2-Dichloro-1-hydroxy) ethyl glutathione | 419.0371 | 5.16 | -1.62 | 2.9×10^-5^ | -0.84 | 1.72 |
| ADP | 427.0267 | 4.80 | -1.51 | 6.2×10^-4^ | -0.76 | 1.12 |
| GDP | 443.0249 | 3.11 | -1.40 | 3.0×10^-5^ | -0.82 | 1.70 |
| UTP | 483.9679 | 4.04 | -2.64 | 4.1×10^-5^ | -0.84 | 1.73 |
| ATP | 506.9938 | 17.18 | -4.18 | 6.2×10^-4^ | -0.78 | 1.17 |
| GTP | 522.9908 | 4.20 | -3.90 | 2.9×10^-5^ | -0.87 | 1.75 |
| 8-Oxo-GTP | 538.9898 | 4.48 | -1.59 | 2.9×10^-5^ | -0.86 | 1.75 |
| UDP-glucose | 566.0534 | 2.76 | -1.92 | 5.1×10^-5^ | -0.91 | 1.31 |
| UDP-glucuronic acid | 580.0338 | 2.35 | -2.10 | 2.9×10^-5^ | -0.84 | 1.72 |
| Ferrocytochrome | 604.174 | 7.96 | -3.04 | 2.3×10^-6^ | -0.97 | 1.55 |
| UDP-N-acetyl-glucosamine | 607.0796 | 4.42 | -1.85 | 4.4×10^-5^ | -0.92 | 1.32 |
| Gluthathione disulfide | 612.1496 | 4.24 | -1.48 | 7.8×10^-4^ | -0.87 | 1.24 |
| Nicotinamide adenine dinucleotide | 663.1087 | 3.29 | -1.39 | 7.6×10^-6^ | -0.84 | 1.40 |
| Trypanothione disulfide | 721.2892 | 8.03 | ↑^a^ |  | 0.02 | 0.73 |

^a^ These metabolites were only found in infected macrophages and therefore it was not possible to calculate log2FC and statistics

Supplementary table 3.

Annotation of metabolites determined as significant (p FDR < 0.05) in the data set of *La*-infected THP-1 treated with miR-372/373/520d inhibitors versus *La*-infected THP-1 treated with negative control (NC) oligonucleotide, referred to as miR × NC.; RSD, relative standard deviation; FC, fold change; FDR, false discovery rate; P(corr), partial correlation coefficient; VIP, variable importance in projection.

| Name | Mass | RSD | Log2FC | p FDR | P(corr) | VIP |
| --- | --- | --- | --- | --- | --- | --- |
| Pyruvic acid | 88.0162 | 2.73 | 0.86 | 7.2×10^-3^ | 0.63 | 1.06 |
| Putrescine | 88.0995 | 7.52 | -0.75 | 6.3×10^-7^ | -0.78 | 1.41 |
| Alanine | 89.0479 | 2.91 | -0.78 | 6.8×10^-5^ | -0.67 | 1.44 |
| Serine | 105.0424 | 4.17 | -0.37 | 7.7×10^-3^ | -0.50 | 0.89 |
| Proline | 115.0633 | 3.75 | -0.55 | 2.1×10^-3^ | -0.58 | 1.20 |
| Valine/Norvaline/Betaine | 117.0790 | 3.46 | -0.52 | 8.0×10^-3^ | -0.50 | 1.02 |
| Succinic acid | 118.0266 | 6.13 | -0.49 | 1.2×10^-2^ | -0.61 | 1.06 |
| Purine | 120.0430 | 12.91 | -1.25 | 7.4×10^-3^ | -0.62 | 1.52 |
| 1-Pyrroline-3-hydroxy-5-carboxylate | 129.0426 | 2.98 | -0.52 | 2.8×10^-2^ | -0.50 | 1.27 |
| trans-4-hydroxyproline | 131.0584 | 3.19 | -0.56 | 4.1×10^-3^ | -0.52 | 1.23 |
| Isoleucine | 131.0946 | 4.72 | -0.86 | 3.2×10^-3^ | -0.65 | 1.65 |
| Leucine | 131.0946 | 3.74 | -0.70 | 5.3×10^-3^ | -0.60 | 1.53 |
| Asparagine | 132.0532 | 3.68 | -0.47 | 3.8×10^-3^ | -0.55 | 1.12 |
| Ornithine | 132.0900 | 3.87 | -0.64 | 6.8×10^-5^ | -0.72 | 1.28 |
| Malic acid | 134.0215 | 4.89 | -0.40 | 2.9×10^-2^ | -0.57 | 1.02 |
| Urocanic acid | 138.0425 | 3.88 | 0.98 | 1.7×10^-2^ | 0.51 | 1.27 |
| Ethanolamine phosphate | 141.0190 | 12.51 | -0.58 | 4.1×10^-3^ | -0.52 | 1.17 |
| Glutamine | 146.0680 | 3.15 | -0.74 | 4.4×10^-3^ | -0.57 | 1.50 |
| Lysine | 146.1051 | 4.67 | -0.36 | 1.4×10^-2^ | -0.46 | 0.97 |
| Methionine | 149.0513 | 12.12 | -0.54 | 1.3×10^-3^ | -0.57 | 1.18 |
| Histidine | 155.0691 | 4.74 | -0.67 | 3.2×10^-4^ | -0.71 | 1.33 |
| 2-Aminoadipic acid | 161.0684 | 10.15 | -0.98 | 1.4×10^-2^ | -0.48 | 1.51 |
| Carnitine | 162.1122 | 3.49 | -0.56 | 5.9×10^-3^ | -0.52 | 1.17 |
| Phenylalanine | 165.0805 | 4.83 | -0.64 | 8.4×10^-4^ | -0.47 | 1.33 |
| Dihydroxyacetone phosphate/  Glyceraldehyde 3-phosphate | 169.9979 | 8.09 | -1.32 | 6.8×10^-3^ | -0.70 | 1.15 |
| Glycerol 1-Phosphate | 172.0131 | 6.10 | -0.84 | 2.4×10^-3^ | -0.76 | 1.19 |
| Arginine | 174.1110 | 2.76 | -0.66 | 3.1×10^-4^ | -0.73 | 1.34 |
| Citrulline | 175.0954 | 18.81 | -0.63 | 5.8×10^-3^ | -0.52 | 1.23 |
| Tyrosine | 181.0749 | 5.94 | -0.46 | 4.1×10^-3^ | -0.53 | 1.07 |
| Mannitol | 182.0793 | 3.79 | -0.77 | 2.4×10^-2^ | -0.48 | 1.32 |
| 2-phosphoglyceric acid/  3-phosphoglyceric acid | 185.9948 | 17.95 | ↑^a^ |  | -0.90 | 1.35 |
| N1-Acetylspermidine | 187.1677 | 6.15 | 1.65 | 1.4×10^-7^ | 0.89 | 2.12 |
| NG,NG-dimethyl-arginine | 202.1428 | 3.77 | -0.65 | 4.2×10^-2^ | -0.41 | 1.15 |
| Spermine | 202.2149 | 3.60 | -0.79 | 4.4×10^-3^ | -0.46 | 1.29 |
| Acetylcarnitine | 203.1153 | 6.05 | -1.06 | 6.7×10^-6^ | -0.73 | 1.67 |
| Tryptophan | 204.0873 | 8.48 | -0.61 | 4.1×10^-3^ | -0.58 | 1.27 |
| Kynurenine | 208.0838 | 25.76 | -1.97 | 2.5×10^-4^ | -0.77 | 2.30 |
| Propionylcarnitine | 217.1311 | 4.58 | -0.98 | 3.2×10^-4^ | -0.52 | 1.33 |
| Cystathionine | 222.0728 | 4.64 | -1.04 | 3.8×10^-4^ | -0.60 | 1.64 |
| Ribulose 5-Phosphate | 230.0188 | 4.69 | -0.82 | 1.6×10^-3^ | -0.68 | 1.13 |
| Cytidine | 243.0872 | 4.68 | 0.68 | 3.4×10^-2^ | 0.43 | 1.03 |
| Glycero-3-Phosphocholine | 257.1026 | 3.56 | -0.61 | 7.4×10^-4^ | -0.61 | 1.26 |
| Cyclic argininosuccinic acid derivative 1 | 272.1130 | 3.87 | -1.15 | 1.8×10^-5^ | -0.76 | 1.73 |
| Sedoheptulose-7-Phosphate | 290.0385 | 4.52 | -0.85 | 2.4×10^-3^ | -0.75 | 1.21 |
| Glutathione | 307.0831 | 15.60 | -0.82 | 2.9×10^-2^ | -0.59 | 0.98 |
| 5'-Methylthioadenosine | 297.0902 | 2.59 | -0.60 | 1.8×10^-2^ | -0.45 | 1.20 |
| Fructose  1,6-bisphosphate | 339.9915 | 8.21 | -1.17 | 5.0×10^-3^ | -0.72 | 1.17 |
| AMP | 347.0630 | 2.11 | -0.58 | 2.6×10^-3^ | -0.58 | 1.40 |
| S-Adenosylmethionine | 398.1379 | 4.08 | -0.49 | 3.4×10^-2^ | -0.39 | 1.05 |
| GDP | 443.0249 | 3.11 | -0.53 | 1.9×10^-2^ | -0.39 | 1.16 |
| ATP | 506.9938 | 17.18 | -2.16 | 1.4×10^-3^ | -0.93 | 1.39 |
| GTP | 522.9908 | 4.20 | -2.14 | 4.4×10^-3^ | -0.52 | 1.37 |
| Ferrocytochrome | 604.1740 | 7.96 | -1.30 | 1.8×10^-3^ | -0.62 | 1.40 |
| UDP-N-acetylglucosamine | 607.0796 | 4.42 | -0.83 | 1.4×10^-3^ | -0.77 | 1.23 |
| Gluthathione disulfide | 612.1496 | 4.24 | -1.24 | 6.2×10^-4^ | -0.85 | 1.27 |
| Trypanothione disulfide | 721.2892 | 8.03 | -0.99 | 2.1×10^-4^ | -0.72 | 1.55 |

^a^ These metabolites were only found in the miR group and therefore it was not possible to calculate log2FC and statistics.

Supplementary Table 4. Gene list for miR-302/372/373/520 family targets retrieved from TargetScan Human 7.2 database.
